# Incidence of admission ionised hypocalcaemia in paediatric major trauma: protocol for a systematic review and meta-analysis

**DOI:** 10.1136/bmjopen-2023-077429

**Published:** 2023-11-10

**Authors:** Owen Hibberd, James Price, Tim Harris, Ed Benjamin Graham Barnard

**Affiliations:** 1 Emergency and Urgent Care Research in Cambridge (EUReCa), PACE Section, Department of Medicine, Cambridge University, Cambridge, UK; 2 Queen Mary University of London, Blizard Institute, London, UK; 3 Department of Research, Audit, Innovation, and Development (RAID), East Anglian Air Ambulance, Norwich, UK; 4 Academic Department of Military Emergency Medicine, Royal Centre for Defence Medicine, Birmingham, UK

**Keywords:** accident & emergency medicine, paediatric intensive & critical care, paediatric A&E and ambulatory care, paediatric orthopaedic & trauma surgery, calcium & bone

## Abstract

**Introduction:**

Hypocalcaemia forms part of the ‘diamond of death’ in major trauma, alongside hypothermia, acidosis and coagulopathy. In adults, admission hypocalcaemia prior to transfusion is associated with increased mortality, increased blood transfusion requirements and coagulopathy. Data on paediatric major trauma patients are limited. This systematic review and meta-analysis aims to describe and synthesise the available evidence relevant to paediatric trauma, admission hypocalcaemia and outcome.

**Methods and analysis:**

The Preferred Reporting Items for Systematic Review and Meta-Analysis Protocols guidelines will be used to construct this review. A planned literature search for articles in the English language will be conducted from inception to the date of searches using MEDLINE on the EBSCO platform, CINAHL on the EBSCO platform and Embase on the Ovid platform. The grey literature will also be searched. Both title and abstract screening and full-text screening will be done by two reviewers, with an adjudicating third reviewer. Heterogeneity will be assessed using the I^2^ test, and the risk of bias will be assessed using the ROBINS-I tool. A meta-analysis will be undertaken using ratio measures (OR) and mean differences for measures of effect. When possible, the estimate of effect will be presented along with a CI and a p value.

**Ethical review and dissemination:**

Ethical review is not required, as no original data will be collected. Results will be disseminated through peer-reviewed publications and at academic conferences.

**PROSPERO registration number:**

CRD42023425172.

**STRENGTHS AND LIMITATIONS OF THIS STUDY**

- The protocol follows the Preferred Reporting Items for Systematic Review and Meta-Analysis Protocols guidelines.
- This is a novel review that addresses an area of uncertainty in the current evidence base surrounding paediatric major trauma through a systematic review and meta-analysis of published data and the grey literature.
- The review methodology is at risk of limitation by publication bias. Where appropriate, this will be assessed using funnel plots.

## Background

Major trauma is one of the leading causes of death in children in the UK.^1 2^

A key cause of potentially survivable death from trauma is haemorrhage.^3^ Uncontrollable haemorrhage may be related to the injury mechanism itself or as a result of trauma-induced coagulopathy (TIC).^4^ TIC is common, occurring in at least a quarter of haemorrhagic deaths, and has a number of proposed pathophysiological mechanisms that generally involve injury and shock, provoking an immunological, endothelial and platelet response.^4^ All forms of haemorrhage are further exacerbated by the ‘lethal triad’ of coagulopathy, hypothermia and acidosis.^5–7^ More recently, biochemical abnormalities such as hyperkalaemia and hypocalcaemia have been recognised to contribute to deaths from haemorrhage.^6 8^ In particular, calcium’s role is important for clot formation, vascular tone and cardiac contractility, with hypocalcaemia contributing to coagulopathy and cardiovascular decompensation.^5 6^ As such, the ‘lethal triad’ is now considered a ‘diamond of death’ with hypocalcaemia forming a key component of this deleterious combination.^5 6^ The early recognition and treatment of these components in the ‘diamond of death’ are essential for trauma resuscitation.^5 7 9^

### Rationale

The free form of calcium (ionised calcium (iCa)) is the physiologically relevant component of calcium in the blood.^10^ iCa is measured on blood gases, which are often taken on arrival for major trauma patients, and there is good agreement between arterial and venous measurements.^11^ Blood gas measurements will also record the pH and lactate, which can affect the availability of iCa.^12 13^ Ionised hypocalcaemia (iHypoCa) in major trauma patients is multifactorial.^5–7 14^ The infusion of citrated blood products is a recognised cause of hypocalcaemia in trauma due to calcium chelation with citrate.^6 15^ There is also emerging evidence in adults that early hypocalcaemia may occur in trauma patients prior to the receipt of blood products containing citrate.^16–19^ Potential pathophysiological mechanisms underpinning this include calcium binding by lactate, the intracellular influx of calcium due to ischaemia and reperfusion, impaired calcium homeostasis secondary to trauma and dilution by crystalloid fluid resuscitation.^5–7^ A systematic review and meta-analysis, which included a total of 1213 major trauma patients, 18 years or older, with a documented iCa level on admission, explored the incidence and outcomes associated with admission iHypoCa.^20^ Studies that involved patients whose calcium concentration may have been confounded by prior blood transfusions were excluded.^20^ Overall, the incidence of admission ionised hypocalcaemia (iHypoCa) was 56.2%, and iHypoCa was associated with increased mortality, increased blood transfusion requirements and coagulopathy.^16–20^ Evidence of admission iHypoCa and the association with adverse outcomes in adult trauma patients has led to the early measurement and replacement of calcium being recommended in adult trauma guidelines.^21 22^ Paediatric major trauma data are limited. Given the different physiology of children compared with adults, children may be more vulnerable to the effects of iHypoCa, and the results of studies involving adult major trauma patients may not be able to be extrapolated to a paediatric cohort.^23^ A search of PROSPERO did not find any similar planned systematic reviews or meta-analyses. Moreover, a preliminary search of the literature has found a few heterogeneous studies, which indicate that admission iHypoCa may be less prevalent in children compared with adults.^24–27^

### Aims

The primary aim of this systematic review and meta-analysis is to explore the limited evidence related to the incidence of admission iHypoCa in paediatric major trauma patients. The review also aims to explore whether admission iHypoCa, compared with normocalcaemia, is associated with adverse clinical outcomes.

## Methods

### Eligibility criteria

This proposed systematic review and meta-analysis will explore the incidence of iHypoCa in paediatric (<18 years old) major trauma patients (Injury Severity Score (ISS) >15) and explore whether admission iHypoCa (iCa <1.16 mmol/L), compared with normocalcaemia (iCa ≥1.16 mmol/L) is associated with a greater incidence of adverse outcomes.^12^ An iCa of <1.16 mmol/L was chosen to reflect different levels of hypocalcaemia thresholds across the literature and facilitate the inclusion of all relevant studies.^24–26^ The Population, Intervention, Comparison, Outcomes and Study Design (PICOS) eligibility criteria are detailed in table 1.

**Table 1:** PICOS strategy for inclusion and exclusion

PICOS strategy	Inclusion criteria	Exclusion criteria
P	Paediatric (<18 years) major trauma patients (injury severity score >15) with a documented iCa level on admission.	iCa level taken after the administration of blood products in the emergency department.
I	Hypocalcaemia on admission (iCa <1.16 mmol/L)	N/A
C	Normocalcaemia on admission (iCa ≥1.16 mmol/L)	N/A
O	Primary outcome include the incidence of admission ionised hypocalcaemia. Secondary outcomes include the association with physiological abnormalities: Haemodynamic instability Hyperkalaemia Hyperlactataemia pH abnormalities Coagulopathy and adverse outcomes: Vasopressor requirement within 24 hours Transfusion requirement within 24 hours Activation of the major haemorrhage protocol within 24 hours Requiring invasive (operative or interventional radiology) intervention within 24 hours Hospital length of stay Paediatric Intensive Care Unit length of stay Early mortality within 24 hours and medium mortality during episode of hospital admission (>24 hours) or within 30 days	N/A
S	Clinical trials (randomised and non-randomised), observational studies (cohort and case-controlled) case reports, case series and literature reviews.	Systematic reviews. Opinion articles.

iCa, ionised calcium; PICOS, Population, Intervention, Comparison, Outcomes, and Study Design.

### Information sources

A planned literature search for articles in the English language will be conducted from inception to the search date using MEDLINE on the EBSCO platform, CINAHL on the EBSCO platform and Embase on the Ovid platform. The reference lists of all included studies and the grey literature will also be searched.

### Search strategy

The search strategy can be found in online supplemental tables 1-3.

10.1136/bmjopen-2023-077429.supp1 Supplementary data

The search will also involve checking reference lists of retrieved articles, conference abstracts and online study results. If the data are incomplete, then the corresponding authors will be contacted for additional information.

### Study records

The search strategy will be undertaken by a trained librarian and information specialist. The combined abstracts from the search strategy will be independently screened by two reviewers to identify studies meeting inclusion criteria; any duplications will be removed manually. For abstracts meeting inclusion criteria, full texts will be retrieved and again independently reviewed against the inclusion and exclusion criteria by two reviewers and an adjudicating third reviewer.

A standardised data sheet (Microsoft Excel for Mac, V.16.72, 2023) will be used to extract data from included studies to facilitate data synthesis and assessment of quality and risk of bias. The extracted data will be independently verified by the second reviewer, and any discrepancies again be adjudicated by the third reviewer.

The following data items will be extracted:

1. Hospital setting.
2. Study type.
3. Country of treatment.
4. Cohort size.
5. ISS.
6. Abbreviated Injury Scale score for injury regions.
7. Ionised hypocalcaemia definitions.
8. Incidence of admission iHypoCa (iCa<1.16 mmol/L).^12^
9. Definitions and presence of coagulopathy
10. The presence of hyperkalaemia (>5.5 mmol/L)^28^
11. The presence of hyperlactataemia (>2.0 mmol/L)^29^
12. Haemodynamic instability (hypotension (based on age-specific Advanced Paediatric Life Support (APLS) values) or elevated Shock Index Paediatric Age-Adjusted (SIPA)).^30–32^
13. Administration of exogenous calcium.
14. Vasoactive medication requirements within the first 24 hours.
15. Total blood product transfusion requirement during the first 24 hours.
16. Activation of the major haemorrhage protocol within the first 24 hours.
17. Requirement for invasive (operative or interventional radiology) intervention within 24 hours.
18. Hospital length of stay (LOS) (days).
19. Paediatric Intensive Care Unit (PICU) LOS (days).
20. Early mortality within 24 hours and medium mortality during an episode of hospital admission (>24 hours) or within 30 days.

### Outcomes and prioritisation

The primary outcome of this systematic review and meta-analysis is the overall incidence of admission iHypoCa. Secondary outcomes are the associations with physiological abnormalities and adverse outcomes. Physiological abnormalities are classified dichotomously as the presence of hypotension (based on age-specific APLS values)^30^ or elevated SIPA (0–6 years: >1.22, 7–12 years: >1.00 and 13–16 years: >0.90),^31 32^ hyperkalaemia (>5.5 mmol/L)^28^ and hyperlactataemia (>2.0 mmol/L).^29^ Adverse outcomes are classified dichotomously as the requirement for vasopressors, transfusion, activation of the major haemorrhage protocol or invasive (operative or interventional radiology) intervention in the first 24 hours and mortality within 30 days. Hospital LOS and PICU LOS in days are classified continuously.

Ratio measures (OR) and mean differences will be used for measures of effect. When possible, the estimate of effect will be presented along with a CI and a p value.

### Risk of bias

The risk of bias will be assessed for all included studies. For any randomised controlled trials, the Grading of Recommendations Assessment, Development and Evaluation methodology will be used, and for observational studies, the Risk Of Bias In Non-randomized Studies of Interventions tool will be used.^33 34^

The risk of publication bias will be assessed with funnel plots as appropriate.^35^

### Data synthesis

The data will be synthesised following Preferred Reporting Items for Systematic Review and Meta-Analysis guidelines. Studies will be assessed clinically (PICO) and methodologically (study design, comparability, outcome ascertainment and risk of bias). Given that current evidence is likely to be limited, the minimum number of studies is two. A preliminary search has identified four studies.^24–27^ The I^2^ test will be conducted to determine if the data are suitable for quantitative synthesis.^36^

Meta-analysis of effect estimates is intended and will be displayed using a forest plot. If there is limited evidence for a prespecified comparison, then the haemodynamic instability and vasopressor PICO groups may be combined. Definitions of hypocalcaemia will also be combined if required, providing values are iCa<1.16 mmol/L. Other elements are unlikely to be suitable as contingencies for a combination. If different effect measures are used, attempts will be made to transform the effect measures for meta-analysis.

A narrative synthesis and summary of effect measures (with the use of box-and-whisker plots) will be conducted if heterogeneity is deemed too substantial across studies to allow for meaningful meta-analysis or if there are major concerns about bias from the three reviewers.

Meta-analysis or narrative synthesis of elements will focus on the incidence of hypocalcaemia in paediatric trauma patients and the trend towards adverse outcomes. Subgroup analysis may be undertaken for severe iHypoCa (iCa<1.0 mmol/L).

### Patient and public involvement

None.

## Ethics and dissemination

Ethical review is not required, as no original data will be collected. Results will be disseminated through peer-reviewed publications and at academic conferences.

## Supplementary Material

Reviewer comments

Author's manuscript

## References

[1] Wolfe I, MacFarlane A, Donkin A, et al. Why children die: death in infants, children and young people in the UK. Royal College of Paediatrics and Child Health, 2014. Available: https://www.rcpch.ac.uk/sites/default/files/2017-06/Why%20children%20die%20-%20Death%20in%20infants%2C%20children%20and%20young%20people%20in%20the%20UK%20-%20Part%20A%202014-05.pdf

[2] Elbourne C, Cole E, Marsh S, et al. At risk child: a contemporary analysis of injured children in London and the South east of England: a prospective, Multicentre cohort study. BMJ Paediatr Open 2021;5:e001114. 10.1136/bmjpo-2021-001114 PMC8573663 34796283

[3] Davis JS, Satahoo SS, Butler FK, et al. An analysis of Prehospital deaths: who can we save J Trauma Acute Care Surg 2014;77:213–8. 10.1097/TA.0000000000000292 25058244

[4] Moore EE, Moore HB, Kornblith LZ, et al. Trauma-induced Coagulopathy [published correction appears in NAT Rev dis primers. Nat Rev Dis Primers 2022;7:30. 10.1038/s41572-021-00264-3

[5] Wray JP, Bridwell RE, Schauer SG, et al. The Diamond of death: hypocalcemia in trauma and resuscitation. Am J Emerg Med 2021;41:104–9. 10.1016/j.ajem.2020.12.065 33421674

[6] DeBot M, Sauaia A, Schaid T, et al. Trauma-induced hypocalcemia. Transfusion 2022;62 Suppl 1:S274–80. 10.1111/trf.16959 35748689

[7] Ditzel RM, Anderson JL, Eisenhart WJ, et al. A review of Transfusion- and trauma-induced hypocalcemia: is it time to change the lethal Triad to the lethal diamond J Trauma Acute Care Surg 2020;88:434–9. 10.1097/TA.0000000000002570 31876689

[8] Brohi K, Gruen RL, Holcomb JB. Why are bleeding trauma patients still dying Intensive Care Med 2019;45:709–11. 10.1007/s00134-019-05560-x 30741331

[9] Braasch MC, Turco LM, Cole EM, et al. The evolution of initial-Hemostatic resuscitation and the void of Posthemostatic resuscitation. J Trauma Acute Care Surg 2020;89:597–601. 10.1097/TA.0000000000002576 32826738

[10] Hamroun A, Pekar J-D, Lionet A, et al. Ionized calcium: Analytical challenges and clinical relevance. J Lab Precis Med 2020;5:22. 10.21037/jlpm-20-60

[11] Bilkovski RN, Cannon CM, Adhikari S, et al. Arterial and venous ionized calcium measurements: is there a difference Annals of Emergency Medicine 2004;44:S56. 10.1016/j.annemergmed.2004.07.183

[12] Egi M, Kim I, Nichol A, et al. Ionized calcium concentration and outcome in critical illness. Crit Care Med 2011;39:314–21. 10.1097/CCM.0b013e3181ffe23e 21099425

[13] Mirzazadeh M, Morovat A, James T, et al. Point-of-care testing of electrolytes and calcium using blood gas Analysers: it is time we trusted the results. Emerg Med J 2016;33:181–6. 10.1136/emermed-2015-204669 26396233

[14] Kronstedt S, Roberts N, Ditzel R, et al. Hypocalcemia as a Predictor of mortality and transfusion. A Scoping review of hypocalcemia in trauma and Hemostatic resuscitation. Transfusion 2022;62:S158–66. 10.1111/trf.16965 35748676 PMC9545337

[15] Byerly S, Inaba K, Biswas S, et al. Transfusion-related hypocalcemia after trauma. World J Surg 2020;44:3743–50. 10.1007/s00268-020-05712-x 32734451 PMC7391918

[16] Webster S, Todd S, Redhead J, et al. Ionised calcium levels in major trauma patients who received blood in the emergency Department. Emerg Med J 2016;33:569–72. 10.1136/emermed-2015-205096 26848163

[17] Cherry RA, Bradburn E, Carney DE, et al. Do early ionized calcium levels really matter in trauma patients J Trauma 2006;61:774–9. 10.1097/01.ta.0000239516.49799.63 17033540

[18] Magnotti LJ, Bradburn EH, Webb DL, et al. Admission ionized calcium levels predict the need for multiple transfusions: a prospective study of 591 critically ill trauma patients. J Trauma 2011;70:391–5; 10.1097/TA.0b013e31820b5d98 21307739

[19] Vasudeva M, Mathew JK, Fitzgerald MC, et al. Hypocalcaemia and traumatic Coagulopathy: an observational analysis. Vox Sang 2020;115:189–95. 10.1111/vox.12875 31845341

[20] Vasudeva M, Mathew JK, Groombridge C, et al. Hypocalcemia in trauma patients: A systematic review. J Trauma Acute Care Surg 2021;90:396–402. 10.1097/TA.0000000000003027 33196630 PMC7850586

[21] Rossaint R, Afshari A, Bouillon B, et al. The European guideline on management of major bleeding and Coagulopathy following trauma. Crit Care 2023;27:80. 10.1186/s13054-023-04327-7 36859355 PMC9977110

[22] Henry S. ATLS 10th edition offers new insights into managing trauma patients Bulletin of the American college of Surgeons. Am Coll Surg 2018:56. Available: https://bulletin.facs.org/2018/06/atls-10th-edition-offers-new-insights-into-managing-traumapatients/#Chapter_3_Shock

[23] Barcelona SL, Thompson AA, Coté CJ. Intraoperative pediatric blood transfusion therapy: a review of common issues. part I: hematologic and physiologic differences from adults; metabolic and infectious risks. Paediatr Anaesth 2005;15:716–26. 10.1111/j.1460-9592.2005.01548.x 16101701

[24] Ciaraglia A, Lumbard D, Deschner B, et al. The effects of hypocalcemia in severely injured pediatric trauma patients. J Trauma Acute Care Surg 2023;95:313–8. 10.1097/TA.0000000000003902 36787433

[25] Gimelraikh Y, Berant R, Stein M, et al. Early hypocalcemia in pediatric major trauma: A retrospective cohort study. Pediatr Emerg Care 2022;38:e1637–40. 10.1097/PEC.0000000000002719 35413033

[26] Epstein D, Ben Lulu H, Raz A, et al. Admission hypocalcemia in pediatric major trauma patients-an uncommon phenomenon associated with an increased need for urgent blood transfusion. Transfusion 2022;62:1341–6. 10.1111/trf.16936 35638746

[27] Cornelius BG, Clark D, Williams B, et al. A retrospective analysis of calcium levels in pediatric trauma patients. Int J Burns Trauma 2021;11:267–74. 34336394 PMC8310872

[28] Wolf J, Geneen LJ, Meli A, et al. Hyperkalaemia following blood transfusion-a systematic review assessing evidence and risks. Transfus Med Rev 2022;36:133–42. 10.1016/j.tmrv.2022.04.003 35534372

[29] Rishu AH, Khan R, Al-Dorzi HM, et al. Even mild Hyperlactatemia is associated with increased mortality in critically ill patients. Crit Care 2013;17:R197. 10.1186/cc12891 24025259 PMC4056896

[30] Samuels M, Wieteska S. Advanced Paediatric life support. In: Advanced Paediatric Life Support: A Practical Approach to Emergencies, 6th edn. Manchester, UK: Advanced Paediatric Life Support Group; New Jersey, USA: John Wiley & Sons Ltd, 2016. 10.1002/9781119241225

[31] Linnaus ME, Notrica DM, Langlais CS, et al. Prospective validation of the shock index pediatric-adjusted (SIPA) in blunt liver and spleen trauma: an ATOMAC+ study. J Pediatr Surg 2017;52:340–4. 10.1016/j.jpedsurg.2016.09.060 27717564

[32] Nordin A, Coleman A, Shi J, et al. Validation of the age-adjusted shock index using pediatric trauma quality improvement program data. J Pediatr Surg 2017:S0022-3468(17)30645-0. 10.1016/j.jpedsurg.2017.10.023 29108845

[33] Atkins D, Best D, Briss PA, et al. Grading quality of evidence and strength of recommendations. BMJ 2004;328:1490. 10.1136/bmj.328.7454.1490 15205295 PMC428525

[34] Sterne JA, Hernán MA, Reeves BC, et al. ROBINS-I: a tool for assessing risk of bias in non-randomised studies of interventions. BMJ 2016;355:i4919. 10.1136/bmj.i4919 27733354 PMC5062054

[35] Sterne JAC, Sutton AJ, Ioannidis JPA, et al. Recommendations for examining and interpreting funnel plot asymmetry in meta-analyses of randomised controlled trials. BMJ 2011;343:bmj.d4002. 10.1136/bmj.d4002 21784880

[36] Migliavaca CB, Stein C, Colpani V, et al. Meta-Analys^I^s of prevalence: I2 Statistic and how to deal with heterogeneity. Res Synth Methods 2022;13:363–7. 10.1002/jrsm.1547 35088937

