# Supplementary material for: Incidence of admission ionised hypocalcaemia in paediatric major trauma: protocol for a systematic review and meta-analysis
Source: BMJ Open. 2023 Nov 10;13(11):e077429. doi: 10.1136/bmjopen-2023-077429 (PMC10649369; doi:10.1136/bmjopen-2023-077429)
Supplement: Supplementary data [file bmjopen-2023-077429supp001.pdf]

Online Supplementary Tables

Supplementary Table 1. CINAHL search strategy for a systematic review and meta-analysis exploring the incidence and associated outcomes with admission hypocalcaemia in paediatric major trauma

Search ID#	Search Terms	Last Run Via	Results from 3rd July 2023
**S29**	S25 AND S28	Interface - EBSCOhost Research Databases<br>Search Screen - Advanced Search<br>Database - CINAHL	2
**S28**	S26 OR S27	Interface - EBSCOhost Research Databases<br>Search Screen - Advanced Search<br>Database - CINAHL	95,090
**S27**	AB trauma	Interface - EBSCOhost Research Databases<br>Search Screen - Advanced Search<br>Database - CINAHL	77,482
**S26**	TI trauma	Interface - EBSCOhost Research Databases<br>Search Screen - Advanced Search<br>Database - CINAHL	39,417
**S25**	S23 OR S24	Interface - EBSCOhost Research Databases<br>Search Screen - Advanced Search<br>Database - CINAHL	11
**S24**	AB paediatric hypocalcaemia or pediatric hypocalcemia	Interface - EBSCOhost Research Databases<br>Search Screen - Advanced Search<br>Database - CINAHL	2
**S23**	TI paediatric hypocalcaemia or pediatric hypocalcemia	Interface - EBSCOhost Research Databases<br>Search Screen - Advanced Search<br>Database - CINAHL	9
**S22**	S3 AND S17	Interface - EBSCOhost Research Databases<br>Search Screen - Advanced Search<br>Database - CINAHL	351
**S21**	S3 AND S12	Interface - EBSCOhost Research Databases<br>Search Screen - Advanced Search<br>Database - CINAHL	0
**S20**	S18 OR S19	Interface - EBSCOhost Research Databases<br>Search Screen - Advanced Search<br>Database - CINAHL	19
**S19**	AB admission hypocalcaemia or admission hypocalcemia	Interface - EBSCOhost Research Databases<br>Search Screen - Advanced Search<br>Database - CINAHL	18

**S18**	TI admission hypocalcaemia or admission hypocalcemia	Interface - EBSCOhost Research Databases<br>Search Screen - Advanced Search<br>Database - CINAHL	1
**S17**	S15 OR S16	Interface - EBSCOhost Research Databases<br>Search Screen - Advanced Search<br>Database - CINAHL	835,597
**S16**	AB outcomes	Interface - EBSCOhost Research Databases<br>Search Screen - Advanced Search<br>Database - CINAHL	732,991
**S15**	TI outcomes	Interface - EBSCOhost Research Databases<br>Search Screen - Advanced Search<br>Database - CINAHL	213,098
**S14**	S3 AND S9 AND S12	Interface - EBSCOhost Research Databases<br>Search Screen - Advanced Search<br>Database - CINAHL	0
**S13**	S3 AND S6	Interface - EBSCOhost Research Databases<br>Search Screen - Advanced Search<br>Database - CINAHL	2
**S12**	S10 OR S11	Interface - EBSCOhost Research Databases<br>Search Screen - Advanced Search<br>Database - CINAHL	83
**S11**	AB normocalcaemia or normocalcemia	Interface - EBSCOhost Research Databases<br>Search Screen - Advanced Search<br>Database - CINAHL	78
**S10**	TI normocalcaemia or normocalcemia	Interface - EBSCOhost Research Databases<br>Search Screen - Advanced Search<br>Database - CINAHL	8
**S9**	S7 OR S8	Interface - EBSCOhost Research Databases<br>Search Screen - Advanced Search<br>Database - CINAHL	1,406
**S8**	AB hospital arrival	Interface - EBSCOhost Research Databases<br>Search Screen - Advanced Search<br>Database - CINAHL	1,349
**S7**	TI hospital arrival	Interface - EBSCOhost Research Databases<br>Search Screen - Advanced Search<br>Database - CINAHL	107
**S6**	S4 OR S5	Interface - EBSCOhost Research Databases<br>Search Screen - Advanced Search<br>Database - CINAHL	1,745
**S5**	AB hypocalcaemia	Interface - EBSCOhost Research Databases	1,451

	or hypocalcemia	Search Screen - Advanced Search<br>Database - CINAHL	
**S4**	TI hypocalcaemia or hypocalcemia	Interface - EBSCOhost Research Databases<br>Search Screen - Advanced Search<br>Database - CINAHL	584
**S3**	S1 OR S2	Interface - EBSCOhost Research Databases<br>Search Screen - Advanced Search<br>Database - CINAHL	1,003
**S2**	AB paediatric trauma patients or pediatric trauma patients	Interface - EBSCOhost Research Databases<br>Search Screen - Advanced Search<br>Database - CINAHL	805
**S1**	TI paediatric trauma patients or pediatric trauma patients	Interface - EBSCOhost Research Databases<br>Search Screen - Advanced Search<br>Database - CINAHL	364

Supplementary Table 2. MEDLINE search strategy for a systematic review and meta-analysis exploring the incidence and associated outcomes with admission hypocalcaemia in paediatric major trauma

Search ID#	Search Terms	Last Run Via	Results from 3rd July 2023
S30	S26 AND S29	Interface - EBSCOhost Research Databases<br>Search Screen - Advanced Search<br>Database - MEDLINE	5
S29	S27 OR S28	Interface - EBSCOhost Research Databases<br>Search Screen - Advanced Search<br>Database - MEDLINE	276,599
S28	AB trauma	Interface - EBSCOhost Research Databases<br>Search Screen - Advanced Search<br>Database - MEDLINE	245,191
S27	TI trauma	Interface - EBSCOhost Research Databases<br>Search Screen - Advanced Search<br>Database - MEDLINE	87,100
S26	S24 OR S25	Interface - EBSCOhost Research Databases<br>Search Screen - Advanced Search<br>Database - MEDLINE	38
S25	AB paediatric hypocalcaemia or pediatric hypocalcemia	Interface - EBSCOhost Research Databases<br>Search Screen - Advanced Search<br>Database - MEDLINE	22
S24	TI paediatric hypocalcaemia or pediatric hypocalcemia	Interface - EBSCOhost Research Databases<br>Search Screen - Advanced Search<br>Database - MEDLINE	18

S23	S6 AND S22	Interface - EBSCOhost Research Databases<br>Search Screen - Advanced Search<br>Database - MEDLINE	4
S22	S3 AND S17	Interface - EBSCOhost Research Databases<br>Search Screen - Advanced Search<br>Database - MEDLINE	746
S21	S3 AND S12	Interface - EBSCOhost Research Databases<br>Search Screen - Advanced Search<br>Database - MEDLINE	0
S20	S18 OR S19	Interface - EBSCOhost Research Databases<br>Search Screen - Advanced Search<br>Database - MEDLINE	72
S19	AB admission hypocalcaemia or admission hypocalcemia	Interface - EBSCOhost Research Databases<br>Search Screen - Advanced Search<br>Database - MEDLINE	71
S18	TI admission hypocalcaemia or admission hypocalcemia	Interface - EBSCOhost Research Databases<br>Search Screen - Advanced Search<br>Database - MEDLINE	4
S17	S15 OR S16	Interface - EBSCOhost Research Databases<br>Search Screen - Advanced Search<br>Database - MEDLINE	2,179,197
S16	AB outcomes	Interface - EBSCOhost Research Databases<br>Search Screen - Advanced Search<br>Database - MEDLINE	2,053,450
S15	TI outcomes	Interface - EBSCOhost Research Databases<br>Search Screen - Advanced Search<br>Database - MEDLINE	450,999
S14	S3 AND S9 AND S12	Interface - EBSCOhost Research Databases<br>Search Screen - Advanced Search<br>Database - MEDLINE	0
S13	S3 AND S6	Interface - EBSCOhost Research Databases<br>Search Screen - Advanced Search<br>Database - MEDLINE	5
S12	S10 OR S11	Interface - EBSCOhost Research Databases<br>Search Screen - Advanced Search<br>Database - MEDLINE	870
S11	AB normocalcaemia or normocalcemia	Interface - EBSCOhost Research Databases<br>Search Screen - Advanced Search<br>Database - MEDLINE	837
S10	TI normocalcaemi	Interface - EBSCOhost Research Databases	52

	a or normocalcemia	Search Screen - Advanced Search<br>Database - MEDLINE	
S9	S7 OR S8	Interface - EBSCOhost Research Databases<br>Search Screen - Advanced Search<br>Database - MEDLINE	80,231
S8	AB hospital arrival or hospital admission	Interface - EBSCOhost Research Databases<br>Search Screen - Advanced Search<br>Database - MEDLINE	76,431
S7	TI hospital arrival or hospital admission	Interface - EBSCOhost Research Databases<br>Search Screen - Advanced Search<br>Database - MEDLINE	8,797
S6	S4 OR S5	Interface - EBSCOhost Research Databases<br>Search Screen - Advanced Search<br>Database - MEDLINE	11,987
S5	AB hypocalcaemia or hypocalcemia	Interface - EBSCOhost Research Databases<br>Search Screen - Advanced Search<br>Database - MEDLINE	10,707
S4	TI hypocalcaemia or hypocalcemia	Interface - EBSCOhost Research Databases<br>Search Screen - Advanced Search<br>Database - MEDLINE	3,153
S3	S1 OR S2	Interface - EBSCOhost Research Databases<br>Search Screen - Advanced Search<br>Database - MEDLINE	2,051
S2	AB paediatric trauma patients or pediatric trauma patients	Interface - EBSCOhost Research Databases<br>Search Screen - Advanced Search<br>Database - MEDLINE	1,764
S1	TI paediatric trauma patients or pediatric trauma patients	Interface - EBSCOhost Research Databases<br>Search Screen - Advanced Search<br>Database - MEDLINE	649

Supplementary Table 3. EMBASE search strategy for a systematic review and meta-analysis exploring the incidence and associated outcomes with admission hypocalcaemia in paediatric major trauma

#	Query	Results from 3rd July 2023
1	(paediatric trauma patients or pediatric trauma patients).m_titl.	384
2	(paediatric trauma patients or pediatric trauma patients).mp. [mp=title, abstract, heading word, drug trade name, original title, device manufacturer, drug manufacturer, device trade name, keyword heading word, floating subheading word, candidate term word]	1,172

3	limit 2 to abstracts	1,155
4	1 or 3	1,172
5	(hypocalcaemia or hypocalcemia).m_titl.	3,811
6	(hypocalcaemia or hypocalcemia).mp. [mp=title, abstract, heading word, drug trade name, original title, device manufacturer, drug manufacturer, device trade name, keyword heading word, floating subheading word, candidate term word]	31,406
7	limit 6 to abstracts	26,928
8	5 or 7	27,890
9	(hospital arrival or hospital admission).m_titl.	4,381
10	(hospital arrival or hospital admission).mp. [mp=title, abstract, heading word, drug trade name, original title, device manufacturer, drug manufacturer, device trade name, keyword heading word, floating subheading word, candidate term word]	292,976
11	limit 10 to abstracts	245,800
12	9 or 11	246,388
13	(normocalcaemia or normocalcemia).m_titl.	63
14	(normocalcaemia or normocalcemia).mp. [mp=title, abstract, heading word, drug trade name, original title, device manufacturer, drug manufacturer, device trade name, keyword heading word, floating subheading word, candidate term word]	1,223
15	limit 14 to abstracts	1,213
16	13 or 15	1,223
17	4 and 8 and 12	1
18	4 and 8	6
19	(paediatric hypocalcaemia or pediatric hypocalcemia).m_titl.	2
20	(paediatric hypocalcaemia or pediatric hypocalcemia).mp. [mp=title, abstract, heading word, drug trade name, original title, device manufacturer, drug manufacturer, device trade name, keyword heading word, floating subheading word, candidate term word]	4
21	limit 20 to abstracts	2
22	19 or 21	4
23	(admission hypocalcaemia or admission hypocalcemia).m_titl.	2
24	(admission hypocalcaemia or admission hypocalcemia).mp. [mp=title, abstract, heading word, drug trade name, original title, device manufacturer, drug manufacturer, device trade name, keyword heading word, floating subheading word, candidate term word]	11
25	limit 24 to abstracts	11
26	23 or 25	11
27	4 and 16	0
