# Supplementary material for: Incidence of admission ionised hypocalcaemia in paediatric major trauma: protocol for a systematic review and meta-analysis
Source: BMJ Open. 2023 Nov 10;13(11):e077429. doi: 10.1136/bmjopen-2023-077429 (PMC10649369; doi:10.1136/bmjopen-2023-077429)
Supplement: Author's manuscript [file bmjopen-2023-077429.draft_revisions.pdf]

BMJ Open is committed to open peer review. As part of this commitment we make the peer review history of every article we publish publicly available.

When an article is published we post the peer reviewers' comments and the authors' responses online. We also post the versions of the paper that were used during peer review. These are the versions that the peer review comments apply to.

The versions of the paper that follow are the versions that were submitted during the peer review process. They are not the versions of record or the final published versions. They should not be cited or distributed as the published version of this manuscript.

BMJ Open is an open access journal and the full, final, typeset and author-corrected version of record of the manuscript is available on our site with no access controls, subscription charges or pay-per-view fees (http://bmjopen.bmj.com).

If you have any questions on BMJ Open's open peer review process please email info.bmjopen@bmj.com

BMJ Open

The Incidence of Admission Ionised Hypocalcaemia in Paediatric Major Trauma: Protocol for a Systematic Review and Meta-Analysis

Journal:	*BMJ Open*
Manuscript ID	bmjopen-2023-077429
Article Type:	Protocol
Date Submitted by the Author:	04-Jul-2023

Complete List of Authors:	Hibberd, Owen; Cambridge University Hospitals NHS Foundation Trust, Emergency Department; Cambridge University, School of Clinical Medicine<br>Price, James ; Cambridge University Hospitals NHS Foundation Trust, Emergency Department; East Anglian Air Ambulance, Department of Research, Audit, Innovation, & Development (RAID)<br>Harris, T; Queen Mary University of London Blizard Institute<br>Barnard, Ed; Cambridge University Hospitals NHS Foundation Trust, Emergency Department ; Royal Centre for Defence Medicine, Academic Department of Military Emergency Medicine

Keywords:	ACCIDENT & EMERGENCY MEDICINE, Paediatric intensive & critical care < INTENSIVE & CRITICAL CARE, Paediatric A&E and ambulatory care < PAEDIATRICS, Paediatric orthopaedic & trauma surgery < PAEDIATRIC SURGERY, Calcium & bone < DIABETES & ENDOCRINOLOGY

SCHOLARONE™
Manuscripts

The Incidence of Admission Ionised Hypocalcaemia in Paediatric Major Trauma: Protocol for a Systematic Review and Meta-Analysis

Registration

PROSPERO registration number: CRD42023425172

Authors

Hibberd O [1,2,3]
Price J [1,4]
Harris T [3]
Barnard EBG [1,4,5]

Affiliations

1. Emergency Department, Cambridge University Hospitals NHS Foundation Trust, Cambridge, UK
2. School of Clinical Medicine, Cambridge University, Cambridge UK
3. Blizard Institute, Queen Mary University of London, London, UK
4. Department of Research, Audit, Innovation, & Development (RAID), East Anglian Air Ambulance, Norwich, UK
5. Academic Department of Military Emergency Medicine, Royal Centre for Defence Medicine (Research and Clinical Innovation), Birmingham, UK

Corresponding author

O Hibberd
oh296@cam.ac.uk

Support

There is no funding or sponsorship to declare.

Abstract

Introduction

Hypocalcaemia forms part of the 'diamond of death' in major trauma, alongside hypothermia, acidosis, and coagulopathy. In adults, admission hypocalcaemia prior to transfusion is associated with increased mortality, increased blood transfusion requirements, and coagulopathy. Data in paediatric major trauma patients are limited. This systematic review and meta-analysis aims to describe and synthesise the available evidence relevant to paediatric trauma, admission hypocalcaemia, and outcome.

Methods and analysis

The Preferred Reporting Items for Systematic Review and Meta-Analysis Protocols (PRISMA-P) guideline will be used to construct this review. A planned literature search for articles in the English language will be conducted from inception to the date of searches using MEDLINE on EBSCO platform, CINAHL on EBSCO platform, and Embase on Ovid platform. The grey literature will also be searched. Both title and abstract screening, and full-text screening will be done by two reviewers, with an adjudicating third reviewer. Heterogeneity will be assessed using I^2 test, and risk of bias will be assessed using the ROBINS-I tool. A meta-analysis will be undertaken using ratio measures (odds

For peer review only

ratio) and mean differences for measures of effect. When possible, the estimate of effect will be presented along with a confidence interval and a *p*-value.

Ethical review and dissemination

Ethical review is not required as no original data will be collected. Results will be disseminated through peer-reviewed publication and at academic conferences.

Prospero registration number

CRD42023425172

Strengths and limitations of this study

⇒ The protocol follows the Preferred Reporting Items for Systematic Review and Meta-Analysis Protocols (PRISMA-P) guidelines.
⇒ This is a novel review that addresses an area of uncertainity in the current evidence base surrounding paediatric major trauma through systematic review and meta-analysis of published data and the grey-literature.
⇒ The review methodology is at risk of limitation by publication bias.

Background

Major trauma is one of the leading causes of death in children in the United Kingdom (UK).[1][2] A key cause of potentially-survivable death from trauma is haemorrhage.[3] Uncontrollable haemorrhage may be related to the injury mechanism itself, or as a result of Trauma Induced Coagulopathy (TIC).[4] TIC is common, occurring in at least a quarter of haemorrhagic deaths, and has a number of proposed pathophysiological mechanisms, which generally involve injury and shock provoking a immunological, endothelial, and platelet response.[4] All forms of haemorrhage are further exacerbated by the 'lethal triad' of coagulopathy, hypothermia and acidosis.[5][6][7] More recently biochemical abnormalities such as hyperkalaemia and hypocalcaemia have been recognised to contribute to deaths from haemorrhage.[6][8] In particular, calcium's role is important for clot formation, vascular tone, and cardiac contractility, with hypocalcaemia contributing to coagulopathy and cardiovascular decompensation.[5][6] As such the 'lethal triad' is now considered a 'diamond of death' with hypocalcaemia forming a key component of this deleterious combination.[5][6] The early recognition and treatment of these components in the 'diamond of death' are essential for trauma resuscitation.[5][7][9]

Rationale

The free form of calcium (ionised calcium (iCa)) is the physiologically relevant component of calcium in the blood.[10] iCa is measured on blood gases, which are often taken on arrival for major trauma patients, and there is good agreement between arterial and venous measurements.[11] Blood gas measurement will also record the pH and lactate, and can affect the availability of iCa.[12][13] Ionised hypocalcaemia (iHypoCa) in major trauma patients is multifactorial.[5][6][7][14] The infusion of citrated blood products is a recognised cause of hypocalcaemia in trauma due to calcium chelation with citrate.[6][15] There is also emerging evidence in adults that early hypocalcaemia may occur in trauma patients prior to the receipt of blood products containing citrate.[16][17][18][19] Potential pathophysiological mechanisms underpinning this include calcium binding by lactate, intracellular influx of calcium due to ischaemia and reperfusion, impaired calcium homeostasis secondary to trauma, and secondary to dilution by crystalloid fluid resuscitation.[5][6][7] A systematic review and meta-analysis, which included a total of 1213 major trauma patients, 18 years or older, with a

document iCa level on admission explored the incidence and outcomes associated with admission iHypoCa.[20] Studies that involved patients in whom calcium concentration may have been confounded by prior blood transfusion were excluded.[20] Overall, the incidence of admission ionised hypocalcaemia (iHypoCa) was 56.2%, and iHypoCa was associated with: increased mortality, increased blood transfusion requirements, and coagulopathy.[16][17][18][19][20] Evidence of admission iHypoCa and the association with adverse outcomes in adult trauma patients has led to the early measurement and replacement of calcium being recommended in adult trauma guidelines.[21][22] In paediatric major trauma data are limited. A search of PROSPERO did not find any similar planned systematic reviews or meta-analyses. Moreover, a preliminary search of the literature has found a few heterogenous studies, which indicate that admission iHypoCa may be less prevalent in children compared to adults.[23][24][25][26]

Aims

The primary aim of this systematic review and meta-analysis is to explore the limited evidence related to the incidence of admission iHypoCa in paediatric major trauma patients. The review also aims to explore whether admission iHypoCa, compared to normocalcaemia, is associated with adverse clinical outcomes.

Methods

Eligibility criteria

This proposed systematic review and meta-analysis will explore the incidence of iHypoCa in paediatric (<16 years old) major trauma patients (Injury Severity Score (ISS) >15) and explore whether admission iHypoCa (iCa <1.16 mmol/L), compared to normocalcaemia (iCa ≥ 1.16 mmol/L) is associated with a greater incidence of adverse outcomes.[12] The Population, Intervention, Comparison, Outcomes, and Study Design (PICOS) eligibility criteria are detailed in Table 1.

Information sources

A planned literature search for articles in the English language will be conducted from inception to the search date using MEDLINE on EBSCO platform, CINAHL on EBSCO platform, and Embase on Ovid platform. The reference lists of all included studies, and the grey literature will also be searched.

Search strategy

The search strategy can be found in online supplementary tables 2,3 and 4.

The search will also involve checking reference lists of retrieved articles, conference abstracts, and online study results. If data is incomplete, then corresponding authors will be contacted for additional information.

Study records

The search strategy will be undertaken by a trained librarian. The combined abstracts from the search strategy will be independently screened by two reviewers to identify studies meeting inclusion criteria, any duplications will be removed manually. For abstracts meeting inclusion criteria, full texts will be retrieved and again be independently reviewed against the inclusion and exclusion criteria by two reviewers and an adjudicating third reviewer.

A standardised data sheet (Microsoft ® Excel for Mac, Version 16.72, 2023) will be used to extract data from included studies to facilitate data synthesis and assessment of quality and risk of bias.

For peer review only

Extracted data will be independently verified by the second reviewer, and any discrepancies again adjudicated by the third reviewer.

The following data items will be extracted:

1. Hospital setting
2. Study type
3. Country of treatment
4. Cohort size
5. Median [interquartile range (IQR)] severity of injury (ISS)
6. Abbreviated Injury Scale (AIS) score for injury regions
7. Ionised hypocalcaemia definitions
8. Incidence of admission iHypoCa (iCa <1.16 mmol/L) (yes/no)[12]
9. Definitions, and presence of, coagulopathy
10. The presence of hyperkalaemia (>5.5 mmol/L)(yes/no/not reported)[27]
11. The presence of hyperlactataemia (>2.0mmol/L) (yes/no/not reported)[28]
12. Haemodynamic instability (hypotension (based upon age specific Advanced Paediatric Life Support (APLS) values) or elevated Shock Index Paediatric Age-Adjusted (SIPA))(yes/no/not reported)[29][30][31]
13. Administration of calcium (yes/no/not reported)
14. Vasopressor requirements within the first 24 hours (yes/no/not reported)
15. Total blood product transfusion requirement during the first 24 hours (yes/no/not reported)
16. Activation of the major haemorrhage protocol within the first 24 hours (yes/no/not reported)
17. Requirement for invasive (operative or interventional radiology) intervention within 24 hours (yes/no/not reported)
18. Hospital length of stay (LOS) (days)
19. Paediatric Intensive Care Unit (PICU) LOS (days)
20. Early mortality within 24 hours and medium mortality during episode of hospital admission (>24 hours) or within 30 days (yes/no/not reported)

iew only

Outcomes and prioritisation

The primary outcome of this systematic review and meta-analysis is the overall incidence of admission iHypoCa. Secondary outcomes are the associations with physiological abnormalities, and adverse outcomes. Physiological abnormalities are classified dichotomously as the presence of hypotension (based upon age specific APLS values)[29] or elevated SIPA(0-6yrs >1.22, 7-12yrs > 1.00, 13-16yrs > 0.90)[30][31], hyperkalaemia (>5.5 mmol/L)[27], and hyperlactataemia (>2.0mmol/L)[28]. Adverse outcomes are classified dichotomously as the requirement for vasopressors, transfusion, activation of the major haemorrhage protocol, or invasive (operative or interventional radiology) intervention in the first 24 hours and mortality within 30 days. Hospital LOS, and PICU LOS in days are classified continuously.

Ratio measures (odds ratio) and mean differences will be used for measures of effect. When possible, the estimate of effect will be presented along with a confidence interval and a *p*-value.

Risk of bias

Risk of bias will be assessed for all included studies. For any randomised controlled trials GRADE methodology will be used, and for observational studies, the ROBINS-I tool will be used.[32][33]

The risk of publication bias will be assessed with funnel plots as appropriate.[36]

Data synthesis

Data will be synthesised following PRISMA guidelines. Studies will be assessed clinically (PICO) and methodologically (study design, comparability, outcome ascertainment, and risk of bias). Given that current evidence is likely to be limited, the minimum number of studies is two. A preliminary search has identified four studies.[23][24][25][26] The I^2 test will be conducted to determine if data are suitable for quantitative synthesis.[34]

Meta-analysis of effect estimates is intended and will be displayed using a forest plot. If there is limited evidence for pre-specified comparison, then the haemodynamic instability and vasopressor PICO groups may be combined. Definitions of hypocalcaemia will also be combined if required, providing values are iCa <1.16. Other elements are unlikely to be suitable as contingencies for combination. If different effect measures are used attempts will be made to transform the effect measures for meta-analysis.

A narrative synthesis and summary of effect measures (with the use of box-and-whisker plots) will be conducted if heterogeneity is deemed too substantial across studies to allow for meaningful meta-analysis or if there are major concerns about bias from the three reviewers.

Meta-analysis or narrative synthesis of elements will focus on the incidence of hypocalcaemia in paediatric trauma patients and trend towards adverse outcomes. Subgroup analysis may be undertaken for severe iHypoCa (iCa <1mmol/L).

Patient and public involvement

No patients will be directly involved in the design of this study and dissemination of findings to participants is not applicable.

Ethics and Dissemination

Ethical review is not required as no original data will be collected. Results will be disseminated through peer-reviewed publication and at academic conferences.

Discussion

The study has the advantage of using rigorous methodology in accordance with the Cochrane handbook and will be reported as per PRISMA guidelines. The methodology is at risk of publication bias.

As paediatric trauma remains a leading cause of death in the UK this study will add to the knowledge base on the management of paediatric major trauma. Given the different physiology of children compared to adults, children may be more vulnerable to the effects of iHypoCa and the results of studies involving adult major trauma patients may not be able to be extrapolated to a paediatric cohort.[35] This study aims to provide further knowledge on the incidence and association of admission hypocalcaemia in paediatric major trauma. Findings of a high incidence of hypocalcaemia, and adverse outcomes, may prompt the consideration of early calcium testing and early replacement for injured children.

Acknowledgements

The authors would like to acknowledge and thank Catherine Hancox and the Defence Medical Academic Library Team for their assistance with the search strategy.

For peer review only

Tables

Table 1. Population, Intervention, Comparison, Outcomes, and Study Design (PICOS) Strategy for Inclusion and Exclusion

PICOS Strategy	Inclusion Criteria	Exclusion Criteria
P – Population	Paediatric (<16yrs) major trauma patients (Injury Severity Score> 15) with a documented ionised calcium (iCa) level on admission.	iCa level taken after the administration of blood products.
I – Intervention	Hypocalcaemia on admission (iCa <1.16 mmol/L)	N/A
C – Comparator	Normocalcaemia on admission (iCa ≥1.16 mmol/L)	N/A
O – Outcome	Primary outcome, the incidence of admission ionised hypocalcaemia.<br><br>Secondary outcomes include the association with physiological abnormalities:<br>▪ Haemodynamic instability<br>▪ Hyperkalaemia<br>▪ Hyperlactataemia<br><br>and adverse outcomes:<br>▪ Vasopressor requirement within 24 hours<br>▪ Transfusion requirement within 24 hours<br>▪ Activation of the major haemorrhage protocol within 24 hours<br>▪ Requiring invasive (operative or interventional radiology) intervention within 24 hours<br>▪ Hospital length of stay<br>▪ PICU length of stay<br>▪ Early mortality within 24 hours and medium mortality during episode of hospital admission (>24 hours) or within 30 days	N/A
S – Study design	Clinical trials (randomised and non-randomised), observational studies (cohort and case-controlled) case	Systematic reviews.<br><br>Opinion articles.

	reports, case series and literature reviews.	

References

1. Wolfe I, MacFarlane A, Donkin A, et al. Why children die: death in infants, children and young people in the UK. Royal College of Paediatrics and Child Health. (2014). Accessed: October 23rd 2022: https://www.rcpch.ac.uk/sites/default/files/2017-06/Why%20children%20die%20-%20Death%20in%20infants%2C%20children%20and%20young%20people%20in%20the%20UK%20-%20Part%20A%202014-05.pdf
2. Elbourne C, Cole E, Marsh S, et al. At risk child: a contemporary analysis of injured children in London and the South East of England: a prospective, multicentre cohort study. BMJ Paediatr Open. 2021;5(1):e001114. Published 2021 Nov 5. doi:10.1136/bmjpo-2021-001114
3. Davis JS, Satahoo SS, Butler FK, et al. An analysis of prehospital deaths. J Trauma Acute Care. 2014;77(2):213–8
4. Moore EE, Moore HB, Kornblith LZ, et al. Trauma-induced coagulopathy [published correction appears in Nat Rev Dis Primers. 2022 Apr 22;8(1):25]. Nat Rev Dis Primers. 2021;7(1):30. Published 2021 Apr 29. doi:10.1038/s41572-021-00264-3
5. Wray JP, Bridwell RE, Schauer SG, et al. The diamond of death: Hypocalcemia in trauma and resuscitation. Am J Emerg Med. 2021;41:104-109. doi:10.1016/j.ajem.2020.12.065
6. DeBot M, Sauaia A, Schaid T, Moore EE. Trauma-induced hypocalcemia. Transfusion. 2022;62 Suppl 1:S274-S280. doi:10.1111/trf.16959
7. Ditzel RM Jr, Anderson JL, Eisenhart WJ, et al. A review of transfusion- and trauma-induced hypocalcemia: Is it time to change the lethal triad to the lethal diamond?. J Trauma Acute Care Surg. 2020;88(3):434-439. doi:10.1097/TA.0000000000002570
8. Brohi K, Gruen RL, Holcomb JB. Why are bleeding trauma patients still dying?. Intensive Care Med. 2019;45(5):709-711. doi:10.1007/s00134-019-05560-x
9. Braasch MC, Turco LM, Cole EM, et al. The evolution of initial-hemostatic resuscitation and the void of posthemostatic resuscitation. J Trauma Acute Care Surg. 2020;89(3):597-601. doi:10.1097/TA.0000000000002576
10. Hamroun A, Pekar JD, Lionet A, et al. Ionized calcium: analytical challenges and clinical relevance. J Lab Precis Med. 2020; 5: 22. http://dx.doi.org/10.21037/jlpm-20-60
11. Bilkovski RN, Cannon CM, Adhikari I, et al. Arterial and venous ionized calcium measurements: Is there a difference? Ann Emerg Med. 2004; 44 (4): S56. https://doi.org/10.1016/j.annemergmed.2004.07.183
12. Egi M, Kim I, Nichol A, et al. Ionized calcium concentration and outcome in critical illness. Crit Care Med. 2011;39:314–321.
13. Mirzazadeh M, Morovat A, James T, et al. Point-of-care testing of electrolytes and calcium using blood gas analysers: it is time we trusted the results. Emerg Med J. 2016;33(3):181-186. doi:10.1136/emermed-2015-204669
14. Kronstedt S, Roberts N, Ditzel R, et al. Hypocalcemia as a predictor of mortality and transfusion. A scoping review of hypocalcemia in trauma and hemostatic resuscitation. Transfusion. 2022;62 Suppl 1(Suppl 1):S158-S166. doi:10.1111/trf.16965
15. Byerly, S., Inaba, K., Biswas, S, et al. Transfusion-Related Hypocalcemia After Trauma. World journal of surgery. 2020;44(11): 3743–3750. https://doi.org/10.1007/s00268-020-05712-x
16. Webster S, Todd S, Redhead J, Wright C. Ionised calcium levels in major trauma patients who received blood in the Emergency Department. Emerg Med J. 2016;33(8):569.
17. Cherry RA, Bradburn E, Carney DE, et al. Do early ionized calcium levels really matter in trauma patients?. J Trauma. 2006;61(4):774-779. doi:10.1097/01.ta.0000239516.49799.63

For peer review only

18. Magnotti LJ, Bradburn EH, Webb DL, et al. Admission ionized calcium levels predict the need for multiple transfusions: a prospective study of 591 critically ill trauma patients. J Trauma. 2011;70(2):391-397. doi:10.1097/TA.0b013e31820b5d98
19. Vasudeva M, Mathew JK, Fitzgerald MC, et al. Hypocalcaemia and traumatic coagulopathy: an observational analysis. Vox Sang. 2020;115(2):189-195. doi:10.1111/vox.12875
20. Vasudeva M, Mathew JK, Groombridge C, et al. Hypocalcemia in trauma patients: A systematic review. J Trauma Acute Care Surg. 2021;90(2):396-402. doi:10.1097/TA.0000000000003027
21. Rossaint, R., Afshari, A., Bouillon, B. et al. The European guideline on management of major bleeding and coagulopathy following trauma: sixth edition. Crit Care. 2023; **27**: 80. https://doi.org/10.1186/s13054-023-04327-7
22. Henry S. ATLS 10th edition offers new insights into managing trauma patients bulletin of the American College of Surgeons. Am Coll Surg. 2018: 56. https://bulletin.facs.org/2018/06/atls-10th-edition-offers-new-insights-into-managing-traumapatients/#Chapter_3_Shock
23. Ciaraglia A, Lumbard D, Deschner B, et al. The Effects of Hypocalcemia in Severely Injured Pediatric Trauma Patients [published online ahead of print, 2023 Feb 13]. J Trauma Acute Care Surg. 2023;10.1097/TA.0000000000003902. doi:10.1097/TA.0000000000003902
24. Gimelraikh Y, Berant R, Stein M, et al. Early Hypocalcemia in Pediatric Major Trauma: A Retrospective Cohort Study [published online ahead of print, 2022 Apr 13]. Pediatr Emerg Care. 2022;10.1097/PEC.0000000000002719. doi:10.1097/PEC.0000000000002719
25. Epstein D, Ben Lulu H, Raz A, Bahouth H. Admission hypocalcemia in pediatric major trauma patients-An uncommon phenomenon associated with an increased need for urgent blood transfusion. Transfusion. 2022;62(7):1341-1346. doi:10.1111/trf.16936
26. Cornelius BG, Clark D, Williams B, et al. A retrospective analysis of calcium levels in pediatric trauma patients. Int J Burns Trauma. 2021;11(3):267-274. Published 2021 Jun 15.
27. Wolf J, Geneen LJ, Meli A, et al. Hyperkalaemia Following Blood Transfusion-a Systematic Review Assessing Evidence and Risks. Transfus Med Rev. 2022;36(3):133-142. doi:10.1016/j.tmrv.2022.04.003
28. Rishu AH, Khan R, Al-Dorzi HM, et al. Even mild hyperlactatemia is associated with increased mortality in critically ill patients. Crit Care. 2013;17(5):R197
29. Samuels M, Wieteska E. Advanced Paediatric Life Support: A Practical Approach to Emergencies, 6th edn. Manchester, UK: Advanced Paediatric Life Support Group; New Jersey, USA: John Wiley & Sons Ltd; 2016. https://doi.org/10.1002/9781119241225
30. Linnaus ME, Notrica DM, Langlais CS, et al. Prospective validation of the shock index pediatric-adjusted (SIPA) in blunt liver and spleen trauma: An ATOMAC+ study. J Pediatr Surg. 2017;52(2):340-344. doi:10.1016/j.jpedsurg.2016.09.060
31. Nordin A, Coleman A, Shi J, et al. Validation of the age-adjusted shock index using pediatric trauma quality improvement program data [published online ahead of print, 2017 Oct 12]. J Pediatr Surg. 2017;S0022-3468(17)30645-0. doi:10.1016/j.jpedsurg.2017.10.023
32. Atkins D, Best D, Briss PA, et al. Grading quality of evidence and strength of recommendations. BMJ. 2004;328(7454):1490. doi:10.1136/bmj.328.7454.1490
33. Sterne JA, Hernán MA, Reeves BC, et al. ROBINS-I: a tool for assessing risk of bias in non-randomised studies of interventions. BMJ. 2016;355:i4919. Published 2016 Oct 12. doi:10.1136/bmj.i4919
34. Migliavaca CB, Stein C, Colpani V, et al. Meta-analysis of prevalence: I^2 statistic and how to deal with heterogeneity. Res Synth Methods. 2022;13(3):363-367. doi:10.1002/jrsm.1547
35. Barcelona SL, Thompson AA, Coté CJ. Intraoperative pediatric blood transfusion therapy: a review of common issues. Part I: hematologic and physiologic differences from adults; metabolic and infectious risks. Paediatr Anaesth. 2005;15(9):716–26.
36. Sterne JA, Sutton AJ, Ioannidis JP, et al. Recommendations for examining and interpreting funnel plot asymmetry in meta-analyses of randomised controlled trials. *BMJ.* 2011;343:d4002. Published 2011 Jul 22. doi:10.1136/bmj.d4002

Online Supplementary Tables

Table 2. CINAHL search strategy for a systematic review and meta-analysis exploring the incidence and associated outcomes with admission hypocalcaemia in paediatric major trauma

Search ID#	Search Terms	Last Run Via	Results from 3rd July 2023
**S29**	S25 AND S28	Interface - EBSCOhost Research Databases<br>Search Screen - Advanced Search<br>Database - CINAHL	2
**S28**	S26 OR S27	Interface - EBSCOhost Research Databases<br>Search Screen - Advanced Search<br>Database - CINAHL	95,090
**S27**	AB trauma	Interface - EBSCOhost Research Databases<br>Search Screen - Advanced Search<br>Database - CINAHL	77,482
**S26**	TI trauma	Interface - EBSCOhost Research Databases<br>Search Screen - Advanced Search<br>Database - CINAHL	39,417
**S25**	S23 OR S24	Interface - EBSCOhost Research Databases<br>Search Screen - Advanced Search<br>Database - CINAHL	11
**S24**	AB paediatric hypocalcaemia or pediatric hypocalcemia	Interface - EBSCOhost Research Databases<br>Search Screen - Advanced Search<br>Database - CINAHL	2
**S23**	TI paediatric hypocalcaemia or pediatric hypocalcemia	Interface - EBSCOhost Research Databases<br>Search Screen - Advanced Search<br>Database - CINAHL	9
**S22**	S3 AND S17	Interface - EBSCOhost Research Databases<br>Search Screen - Advanced Search<br>Database - CINAHL	351
**S21**	S3 AND S12	Interface - EBSCOhost Research Databases<br>Search Screen - Advanced Search<br>Database - CINAHL	0

For peer review only

**S20**	S18 OR S19	Interface - EBSCOhost Research Databases<br>Search Screen - Advanced Search<br>Database - CINAHL	19
**S19**	AB admission hypocalcaemia or admission hypocalcemia	Interface - EBSCOhost Research Databases<br>Search Screen - Advanced Search<br>Database - CINAHL	18
**S18**	TI admission hypocalcaemia or admission hypocalcemia	Interface - EBSCOhost Research Databases<br>Search Screen - Advanced Search<br>Database - CINAHL	1
**S17**	S15 OR S16	Interface - EBSCOhost Research Databases<br>Search Screen - Advanced Search<br>Database - CINAHL	835,597
**S16**	AB outcomes	Interface - EBSCOhost Research Databases<br>Search Screen - Advanced Search<br>Database - CINAHL	732,991
**S15**	TI outcomes	Interface - EBSCOhost Research Databases<br>Search Screen - Advanced Search<br>Database - CINAHL	213,098
**S14**	S3 AND S9 AND S12	Interface - EBSCOhost Research Databases<br>Search Screen - Advanced Search<br>Database - CINAHL	0
**S13**	S3 AND S6	Interface - EBSCOhost Research Databases<br>Search Screen - Advanced Search<br>Database - CINAHL	2
**S12**	S10 OR S11	Interface - EBSCOhost Research Databases<br>Search Screen - Advanced Search<br>Database - CINAHL	83
**S11**	AB normocalcaemia or normocalcemia	Interface - EBSCOhost Research Databases<br>Search Screen - Advanced Search<br>Database - CINAHL	78
**S10**	TI normocalcaemia or normocalcemia	Interface - EBSCOhost Research Databases<br>Search Screen - Advanced	8

		Search<br>Database - CINAHL	
**S9**	S7 OR S8	Interface - EBSCOhost Research Databases<br>Search Screen - Advanced Search<br>Database - CINAHL	1,406
**S8**	AB hospital arrival	Interface - EBSCOhost Research Databases<br>Search Screen - Advanced Search<br>Database - CINAHL	1,349
**S7**	TI hospital arrival	Interface - EBSCOhost Research Databases<br>Search Screen - Advanced Search<br>Database - CINAHL	107
**S6**	S4 OR S5	Interface - EBSCOhost Research Databases<br>Search Screen - Advanced Search<br>Database - CINAHL	1,745
**S5**	AB hypocalcaemia or hypocalcemia	Interface - EBSCOhost Research Databases<br>Search Screen - Advanced Search<br>Database - CINAHL	1,451
**S4**	TI hypocalcaemia or hypocalcemia	Interface - EBSCOhost Research Databases<br>Search Screen - Advanced Search<br>Database - CINAHL	584
**S3**	S1 OR S2	Interface - EBSCOhost Research Databases<br>Search Screen - Advanced Search<br>Database - CINAHL	1,003
**S2**	AB paediatric trauma patients or pediatric trauma patients	Interface - EBSCOhost Research Databases<br>Search Screen - Advanced Search<br>Database - CINAHL	805
**S1**	TI paediatric trauma patients or pediatric trauma patients	Interface - EBSCOhost Research Databases<br>Search Screen - Advanced Search<br>Database - CINAHL	364

Table 3. MEDLINE search strategy for a systematic review and meta-analysis exploring the incidence and associated outcomes with admission hypocalcaemia in paediatric major trauma

Search ID#	Search Terms	Last Run Via	Results from 3rd July 2023

S30	S26 AND S29	Interface - EBSCOhost Research Databases<br>Search Screen - Advanced Search<br>Database - MEDLINE	5
S29	S27 OR S28	Interface - EBSCOhost Research Databases<br>Search Screen - Advanced Search<br>Database - MEDLINE	276,599
S28	AB trauma	Interface - EBSCOhost Research Databases<br>Search Screen - Advanced Search<br>Database - MEDLINE	245,191
S27	TI trauma	Interface - EBSCOhost Research Databases<br>Search Screen - Advanced Search<br>Database - MEDLINE	87,100
S26	S24 OR S25	Interface - EBSCOhost Research Databases<br>Search Screen - Advanced Search<br>Database - MEDLINE	38
S25	AB paediatric hypocalcaemia or pediatric hypocalcemia	Interface - EBSCOhost Research Databases<br>Search Screen - Advanced Search<br>Database - MEDLINE	22
S24	TI paediatric hypocalcaemia or pediatric hypocalcemia	Interface - EBSCOhost Research Databases<br>Search Screen - Advanced Search<br>Database - MEDLINE	18
S23	S6 AND S22	Interface - EBSCOhost Research Databases<br>Search Screen - Advanced Search<br>Database - MEDLINE	4
S22	S3 AND S17	Interface - EBSCOhost Research Databases<br>Search Screen - Advanced Search<br>Database - MEDLINE	746
S21	S3 AND S12	Interface - EBSCOhost Research Databases<br>Search Screen - Advanced Search<br>Database - MEDLINE	0
S20	S18 OR S19	Interface - EBSCOhost Research Databases<br>Search Screen - Advanced Search<br>Database - MEDLINE	72
S19	AB admission hypocalcaemia or admission hypocalcemia	Interface - EBSCOhost Research Databases<br>Search Screen - Advanced Search<br>Database - MEDLINE	71
S18	TI admission hypocalcaemia or admission hypocalcemia	Interface - EBSCOhost Research Databases<br>Search Screen - Advanced Search<br>Database - MEDLINE	4
S17	S15 OR S16	Interface - EBSCOhost Research Databases	2,179,197

For peer review only

		Search Screen - Advanced Search<br>Database - MEDLINE	
S16	AB outcomes	Interface - EBSCOhost Research Databases<br>Search Screen - Advanced Search<br>Database - MEDLINE	2,053,450
S15	TI outcomes	Interface - EBSCOhost Research Databases<br>Search Screen - Advanced Search<br>Database - MEDLINE	450,999
S14	S3 AND S9 AND S12	Interface - EBSCOhost Research Databases<br>Search Screen - Advanced Search<br>Database - MEDLINE	0
S13	S3 AND S6	Interface - EBSCOhost Research Databases<br>Search Screen - Advanced Search<br>Database - MEDLINE	5
S12	S10 OR S11	Interface - EBSCOhost Research Databases<br>Search Screen - Advanced Search<br>Database - MEDLINE	870
S11	AB normocalcaemia or normocalcemia	Interface - EBSCOhost Research Databases<br>Search Screen - Advanced Search<br>Database - MEDLINE	837
S10	TI normocalcaemia or normocalcemia	Interface - EBSCOhost Research Databases<br>Search Screen - Advanced Search<br>Database - MEDLINE	52
S9	S7 OR S8	Interface - EBSCOhost Research Databases<br>Search Screen - Advanced Search<br>Database - MEDLINE	80,231
S8	AB hospital arrival or hospital admission	Interface - EBSCOhost Research Databases<br>Search Screen - Advanced Search<br>Database - MEDLINE	76,431
S7	TI hospital arrival or hospital admission	Interface - EBSCOhost Research Databases<br>Search Screen - Advanced Search<br>Database - MEDLINE	8,797
S6	S4 OR S5	Interface - EBSCOhost Research Databases<br>Search Screen - Advanced Search<br>Database - MEDLINE	11,987
S5	AB hypocalcaemia or hypocalcemia	Interface - EBSCOhost Research Databases<br>Search Screen - Advanced Search<br>Database - MEDLINE	10,707
S4	TI hypocalcaemia or hypocalcemia	Interface - EBSCOhost Research Databases<br>Search Screen - Advanced Search<br>Database - MEDLINE	3,153

For peer review only

S3	S1 OR S2	Interface - EBSCOhost Research Databases<br>Search Screen - Advanced Search<br>Database - MEDLINE	2,051
S2	AB paediatric trauma patients or pediatric trauma patients	Interface - EBSCOhost Research Databases<br>Search Screen - Advanced Search<br>Database - MEDLINE	1,764
S1	TI paediatric trauma patients or pediatric trauma patients	Interface - EBSCOhost Research Databases<br>Search Screen - Advanced Search<br>Database - MEDLINE	649

Table 4. EMBASE search strategy for a systematic review and meta-analysis exploring the incidence and associated outcomes with admission hypocalcaemia in paediatric major trauma

#	Query	Results from 3rd July 2023
1	(paediatric trauma patients or pediatric trauma patients).m_titl.	384
2	(paediatric trauma patients or pediatric trauma patients).mp. [mp=title, abstract, heading word, drug trade name, original title, device manufacturer, drug manufacturer, device trade name, keyword heading word, floating subheading word, candidate term word]	1,172
3	limit 2 to abstracts	1,155
4	1 or 3	1,172
5	(hypocalcaemia or hypocalcemia).m_titl.	3,811
6	(hypocalcaemia or hypocalcemia).mp. [mp=title, abstract, heading word, drug trade name, original title, device manufacturer, drug manufacturer, device trade name, keyword heading word, floating subheading word, candidate term word]	31,406
7	limit 6 to abstracts	26,928
8	5 or 7	27,890
9	(hospital arrival or hospital admission).m_titl.	4,381
10	(hospital arrival or hospital admission).mp. [mp=title, abstract, heading word, drug trade name, original title, device manufacturer, drug manufacturer, device trade name, keyword heading word, floating subheading word, candidate term word]	292,976
11	limit 10 to abstracts	245,800
12	9 or 11	246,388
13	(normocalcaemia or normocalcemia).m_titl.	63
14	(normocalcaemia or normocalcemia).mp. [mp=title, abstract, heading word, drug trade name, original title, device manufacturer, drug manufacturer, device trade name, keyword heading word, floating subheading word, candidate term word]	1,223
15	limit 14 to abstracts	1,213

For peer review only

16	13 or 15	1,223
17	4 and 8 and 12	1
18	4 and 8	6
19	(paediatric hypocalcaemia or pediatric hypocalcemia).m_titl.	2
20	(paediatric hypocalcaemia or pediatric hypocalcemia).mp. [mp=title, abstract, heading word, drug trade name, original title, device manufacturer, drug manufacturer, device trade name, keyword heading word, floating subheading word, candidate term word]	4
21	limit 20 to abstracts	2
22	19 or 21	4
23	(admission hypocalcaemia or admission hypocalcemia).m_titl.	2
24	(admission hypocalcaemia or admission hypocalcemia).mp. [mp=title, abstract, heading word, drug trade name, original title, device manufacturer, drug manufacturer, device trade name, keyword heading word, floating subheading word, candidate term word]	11
25	limit 24 to abstracts	11
26	23 or 25	11
27	4 and 16	0

For peer review only

PRISMA-P (Preferred Reporting Items for Systematic review and Meta-Analysis Protocols) 2015 checklist: recommended items to address in a systematic review protocol*

Section and topic	Item No	Checklist item	
**ADMINISTRATIVE INFORMATION**			
Title:			
Identification	1a	Identify the report as a protocol of a systematic review	Page 1
Update	1b	If the protocol is for an update of a previous systematic review, identify as such	
Registration	2	If registered, provide the name of the registry (such as PROSPERO) and registration number	Page 1
Authors:			
Contact	3a	Provide name, institutional affiliation, e-mail address of all protocol authors; provide physical mailing address of corresponding author	Page 1
Contributions	3b	Describe contributions of protocol authors and identify the guarantor of the review	
Amendments	4	If the protocol represents an amendment of a previously completed or published protocol, identify as such and list changes; otherwise, state plan for documenting important protocol amendments	N/A
Support:			
Sources	5a	Indicate sources of financial or other support for the review	
Sponsor	5b	Provide name for the review funder and/or sponsor	Page 1
Role of sponsor or funder	5c	Describe roles of funder(s), sponsor(s), and/or institution(s), if any, in developing the protocol	
**INTRODUCTION**			
Rationale	6	Describe the rationale for the review in the context of what is already known	Page 2
Objectives	7	Provide an explicit statement of the question(s) the review will address with reference to participants, interventions, comparators, and outcomes (PICO)	Page 3
**METHODS**			
Eligibility criteria	8	Specify the study characteristics (such as PICO, study design, setting, time frame) and report characteristics (such as years considered, language, publication status) to be used as criteria for eligibility for the review	Page 3
Information sources	9	Describe all intended information sources (such as electronic databases, contact with study authors, trial registers or other grey literature sources) with planned dates of coverage	Page 3
Search strategy	10	Present draft of search strategy to be used for at least one electronic database, including planned limits, such that it could be repeated	Page 3
Study records:			
Data management	11a	Describe the mechanism(s) that will be used to manage records and data throughout the review	Page 3

For peer review only

Selection process	11b	State the process that will be used for selecting studies (such as two independent reviewers) through each phase of the review (that is, screening, eligibility and inclusion in meta-analysis)	
Data collection process	11c	Describe planned method of extracting data from reports (such as piloting forms, done independently, in duplicate), any processes for obtaining and confirming data from investigators	Page 3
Data items	12	List and define all variables for which data will be sought (such as PICO items, funding sources), any pre-planned data assumptions and simplifications	
Outcomes and prioritization	13	List and define all outcomes for which data will be sought, including prioritization of main and additional outcomes, with rationale	Page 3
Risk of bias in individual studies	14	Describe anticipated methods for assessing risk of bias of individual studies, including whether this will be done at the outcome or study level, or both; state how this information will be used in data synthesis	Page 3
Data synthesis	15a	Describe criteria under which study data will be quantitatively synthesised	
	15b	If data are appropriate for quantitative synthesis, describe planned summary measures, methods of handling data and methods of combining data from studies, including any planned exploration of consistency (such as I^2, Kendall's τ)	Page 4
	15c	Describe any proposed additional analyses (such as sensitivity or subgroup analyses, meta-regression)	
	15d	If quantitative synthesis is not appropriate, describe the type of summary planned	
Meta-bias(es)	16	Specify any planned assessment of meta-bias(es) (such as publication bias across studies, selective reporting within studies)	Page 4
Confidence in cumulative evidence	17	Describe how the strength of the body of evidence will be assessed (such as GRADE)	Page 4

*** It is strongly recommended that this checklist be read in conjunction with the PRISMA-P Explanation and Elaboration (cite when available) for important clarification on the items. Amendments to a review protocol should be tracked and dated. The copyright for PRISMA-P (including checklist) is held by the PRISMA-P Group and is distributed under a Creative Commons Attribution Licence 4.0.**

From: Shamseer L, Moher D, Clarke M, Ghersi D, Liberati A, Petticrew M, Shekelle P, Stewart L, PRISMA-P Group. Preferred reporting items for systematic review and meta-analysis protocols (PRISMA-P) 2015: elaboration and explanation. BMJ. 2015 Jan 2;349(jan02 1):g7647.

For peer review only

BMJ Open

The Incidence of Admission Ionised Hypocalcaemia in Paediatric Major Trauma: Protocol for a Systematic Review and Meta-Analysis

Journal:	*BMJ Open*
Manuscript ID	bmjopen-2023-077429.R1
Article Type:	Protocol
Date Submitted by the Author:	13-Oct-2023
Complete List of Authors:	Hibberd, Owen; Cambridge University, Emergency and Urgent Care Research in Cambridge (EUReCa), PACE Section, Department of Medicine, Cambridge University, UK<br>Price, James ; Cambridge University, Emergency and Urgent Care Research in Cambridge (EUReCa), PACE Section, Department of Medicine, Cambridge University, UK; East Anglian Air Ambulance, Department of Research, Audit, Innovation, & Development (RAID)<br>Harris, T; Queen Mary University of London Blizard Institute<br>Barnard, Ed; Cambridge University, Emergency and Urgent Care Research in Cambridge (EUReCa), PACE Section, Department of Medicine, Cambridge University, UK; Royal Centre for Defence Medicine, Academic Department of Military Emergency Medicine
**Primary Subject Heading**:	Paediatrics
Secondary Subject Heading:	Emergency medicine, Intensive care, Surgery
Keywords:	ACCIDENT & EMERGENCY MEDICINE, Paediatric intensive & critical care < INTENSIVE & CRITICAL CARE, Paediatric A&E and ambulatory care < PAEDIATRICS, Paediatric orthopaedic & trauma surgery < PAEDIATRIC SURGERY, Calcium & bone < DIABETES & ENDOCRINOLOGY

SCHOLARONE™
Manuscripts

Abstract

Introduction

Hypocalcaemia forms part of the 'diamond of death' in major trauma, alongside hypothermia, acidosis, and coagulopathy. In adults, admission hypocalcaemia prior to transfusion is associated with increased mortality, increased blood transfusion requirements, and coagulopathy. Data in paediatric major trauma patients are limited. This systematic review and meta-analysis aims to describe and synthesise the available evidence relevant to paediatric trauma, admission hypocalcaemia, and outcome.

Methods and analysis

The Preferred Reporting Items for Systematic Review and Meta-Analysis Protocols (PRISMA-P) guideline will be used to construct this review. A planned literature search for articles in the English language will be conducted from inception to the date of searches using MEDLINE on EBSCO platform, CINAHL on EBSCO platform, and Embase on Ovid platform. The grey literature will also be searched. Both title and abstract screening, and full-text screening will be done by two reviewers, with an adjudicating third reviewer. Heterogeneity will be assessed using I^2 test, and risk of bias will be assessed using the ROBINS-I tool. A meta-analysis will be undertaken using ratio measures (odds ratio) and mean differences for measures of effect. When possible, the estimate of effect will be presented along with a confidence interval and a *p*-value.

Ethical review and dissemination

Ethical review is not required as no original data will be collected. Results will be disseminated through peer-reviewed publication and at academic conferences.

Prospero registration number

CRD42023425172

**Strengths and limitations of this study**
⇒ The protocol follows the Preferred Reporting Items for Systematic Review and Meta-Analysis Protocols (PRISMA-P) guidelines.<br>⇒ This is a novel review that addresses an area of uncertainity in the current evidence base surrounding paediatric major trauma through systematic review and meta-analysis of published data and the grey-literature.<br>⇒ The review methodology is at risk of limitation by publication bias. Where appropriate this will be assessed using funnel plots.

Background

Major trauma is one of the leading causes of death in children in the United Kingdom (UK).[1][2] A key cause of potentially-survivable death from trauma is haemorrhage.[3] Uncontrollable haemorrhage may be related to the injury mechanism itself, or as a result of Trauma Induced Coagulopathy (TIC).[4] TIC is common, occurring in at least a quarter of haemorrhagic deaths, and has a number of proposed pathophysiological mechanisms, which generally involve injury and shock provoking a immunological, endothelial, and platelet response.[4] All forms of haemorrhage are further exacerbated by the 'lethal triad' of coagulopathy, hypothermia and acidosis.[5][6][7] More recently biochemical abnormalities such as hyperkalaemia and hypocalcaemia have been recognised

For peer review only

to contribute to deaths from haemorrhage.[6][8] In particular, calcium's role is important for clot formation, vascular tone, and cardiac contractility, with hypocalcaemia contributing to coagulopathy and cardiovascular decompensation.[5][6]] As such the 'lethal triad' is now considered a 'diamond of death' with hypocalcaemia forming a key component of this deleterious combination.[5][6]] The early recognition and treatment of these components in the 'diamond of death' are essential for trauma resuscitation.[5][7][9]

Rationale

The free form of calcium (ionised calcium (iCa)) is the physiologically relevant component of calcium in the blood.[10] iCa is measured on blood gases, which are often taken on arrival for major trauma patients, and there is good agreement between arterial and venous measurements.[11] Blood gas measurement will also record the pH and lactate, and can affect the availability of iCa.[12][13] Ionised hypocalcaemia (iHypoCa) in major trauma patients is multifactorial.[5][6][7][14] The infusion of citrated blood products is a recognised cause of hypocalcaemia in trauma due to calcium chelation with citrate.[6][15] There is also emerging evidence in adults that early hypocalcaemia may occur in trauma patients prior to the receipt of blood products containing citrate.[16][17][18][19] Potential pathophysiological mechanisms underpinning this include calcium binding by lactate, intracellular influx of calcium due to ischaemia and reperfusion, impaired calcium homeostasis secondary to trauma, and secondary to dilution by crystalloid fluid resuscitation.[5][6][7] A systematic review and meta-analysis, which included a total of 1213 major trauma patients, 18 years or older, with a document iCa level on admission explored the incidence and outcomes associated with admission iHypoCa.[20] Studies that involved patients in whom calcium concentration may have been confounded by prior blood transfusion were excluded.[20] Overall, the incidence of admission ionised hypocalcaemia (iHypoCa) was 56.2%, and iHypoCa was associated with: increased mortality, increased blood transfusion requirements, and coagulopathy.[16][17][18][19][20] Evidence of admission iHypoCa and the association with adverse outcomes in adult trauma patients has led to the early measurement and replacement of calcium being recommended in adult trauma guidelines.[21][22] In paediatric major trauma data are limited. Given the different physiology of children compared to adults, children may be more vulnerable to the effects of iHypoCa and the results of studies involving adult major trauma patients may not be able to be extrapolated to a paediatric cohort.[23] A search of PROSPERO did not find any similar planned systematic reviews or meta-analyses. Moreover, a preliminary search of the literature has found a few heterogenous studies, which indicate that admission iHypoCa may be less prevalent in children compared to adults.[24][25][26][27]

Aims

The primary aim of this systematic review and meta-analysis is to explore the limited evidence related to the incidence of admission iHypoCa in paediatric major trauma patients. The review also aims to explore whether admission iHypoCa, compared to normocalcaemia, is associated with adverse clinical outcomes.

Methods

<u>Eligibility criteria</u>

This proposed systematic review and meta-analysis will explore the incidence of iHypoCa in paediatric (<18 years old) major trauma patients (Injury Severity Score (ISS) >15) and explore whether admission iHypoCa (iCa <1.16 mmol/L), compared to normocalcaemia (iCa ≥ 1.16 mmol/L) is associated with a greater incidence of adverse outcomes.[12] An iCa of <1.16 mmol/L was chosen to reflect different levels of hypocalcacemia thresholds across the literature and facilitate inclusion of all relevant studies.[24][25][26] The Population, Intervention, Comparison, Outcomes, and Study Design (PICOS) eligibility criteria are detailed in Table 1.

For peer review only - http://bmjopen.bmj.com/site/about/guidelines.xhtml

Information sources

A planned literature search for articles in the English language will be conducted from inception to the search date using MEDLINE on EBSCO platform, CINAHL on EBSCO platform, and Embase on Ovid platform. The reference lists of all included studies, and the grey literature will also be searched.

Search strategy

The search strategy can be found in online supplementary tables 1,2 and 3.

The search will also involve checking reference lists of retrieved articles, conference abstracts, and online study results. If data is incomplete, then corresponding authors will be contacted for additional information.

Study records

The search strategy will be undertaken by a trained librarian and information specialist. The combined abstracts from the search strategy will be independently screened by two reviewers to identify studies meeting inclusion criteria, any duplications will be removed manually. For abstracts meeting inclusion criteria, full texts will be retrieved and again be independently reviewed against the inclusion and exclusion criteria by two reviewers and an adjudicating third reviewer.

A standardised data sheet (Microsoft ® Excel for Mac, Version 16.72, 2023) will be used to extract data from included studies to facilitate data synthesis and assessment of quality and risk of bias. Extracted data will be independently verified by the second reviewer, and any discrepancies again adjudicated by the third reviewer.

The following data items will be extracted:

1. Hospital setting
2. Study type
3. Country of treatment
4. Cohort size
5. Injury severity score (ISS)
6. Abbreviated Injury Scale (AIS) score for injury regions
7. Ionised hypocalcaemia definitions
8. Incidence of admission iHypoCa (iCa <1.16 mmol/L)[12]
9. Definitions, and presence of, coagulopathy
10. The presence of hyperkalaemia (>5.5 mmol/L)[28]
11. The presence of hyperlactataemia (>2.0mmol/L)[29]
12. Haemodynamic instability (hypotension (based upon age specific Advanced Paediatric Life Support (APLS) values) or elevated Shock Index Paediatric Age-Adjusted (SIPA))[30][31][32]
13. Administration of exogenous calcium
14. Vasoactive medication requirements within the first 24 hours
15. Total blood product transfusion requirement during the first 24 hours
16. Activation of the major haemorrhage protocol within the first 24 hours
17. Requirement for invasive (operative or interventional radiology) intervention within 24 hours
18. Hospital length of stay (LOS) (days)
19. Paediatric Intensive Care Unit (PICU) LOS (days)
20. Early mortality within 24 hours and medium mortality during episode of hospital admission (>24 hours) or within 30 days

For peer review only

Outcomes and prioritisation

The primary outcome of this systematic review and meta-analysis is the overall incidence of admission iHypoCa. Secondary outcomes are the associations with physiological abnormalities, and adverse outcomes. Physiological abnormalities are classified dichotomously as the presence of hypotension (based upon age specific APLS values)[30] or elevated SIPA(0-6yrs >1.22, 7-12yrs > 1.00, 13-16yrs > 0.90),[31][32] hyperkalaemia (>5.5 mmol/L),[28] and hyperlactataemia (>2.0mmol/L).[29] Adverse outcomes are classified dichotomously as the requirement for vasopressors, transfusion, activation of the major haemorrhage protocol, or invasive (operative or interventional radiology) intervention in the first 24 hours and mortality within 30 days. Hospital LOS, and PICU LOS in days are classified continuously.

Ratio measures (odds ratio) and mean differences will be used for measures of effect. When possible, the estimate of effect will be presented along with a confidence interval and a *p*-value.

Risk of bias

Risk of bias will be assessed for all included studies. For any randomised controlled trials GRADE methodology will be used, and for observational studies, the ROBINS-I tool will be used.[33][34]

The risk of publication bias will be assessed with funnel plots as appropriate.[35]

Data synthesis

Data will be synthesised following PRISMA guidelines. Studies will be assessed clinically (PICO) and methodologically (study design, comparability, outcome ascertainment, and risk of bias). Given that current evidence is likely to be limited, the minimum number of studies is two. A preliminary search has identified four studies.[24][25][26][27] The I2 test will be conducted to determine if data are suitable for quantitative synthesis.[36]

Meta-analysis of effect estimates is intended and will be displayed using a forest plot. If there is limited evidence for pre-specified comparison, then the haemodynamic instability and vasopressor PICO groups may be combined. Definitions of hypocalcaemia will also be combined if required, providing values are iCa <1.16 mmol/L. Other elements are unlikely to be suitable as contingencies for combination. If different effect measures are used attempts will be made to transform the effect measures for meta-analysis.

A narrative synthesis and summary of effect measures (with the use of box-and-whisker plots) will be conducted if heterogeneity is deemed too substantial across studies to allow for meaningful meta-analysis or if there are major concerns about bias from the three reviewers.

Meta-analysis or narrative synthesis of elements will focus on the incidence of hypocalcaemia in paediatric trauma patients and trend towards adverse outcomes. Subgroup analysis may be undertaken for severe iHypoCa (iCa <1.0 mmol/L).

Patient and public involvement

None

Ethics and Dissemination

Ethical review is not required as no original data will be collected. Results will be disseminated through peer-reviewed publication and at academic conferences.

Contributorship statement

OH conceptualised the protocol. OH, JP, TH, and EB all contributed to the design, data interpretation, critical revision and final approval of the protocol.

Competing interests

None

Funding

None

Acknowledgements

The authors would like to acknowledge and thank Catherine Hancox and the Defence Medical Academic Library Team for their assistance with the search strategy.

Tables

Table 1. Population, Intervention, Comparison, Outcomes, and Study Design (PICOS) Strategy for Inclusion and Exclusion

PICOS Strategy	Inclusion Criteria	Exclusion Criteria
P – Population	Paediatric (<18yrs) major trauma patients (Injury Severity Score> 15) with a documented ionised calcium (iCa) level on admission.	iCa level taken after the administration of blood products in the Emergency Department.
I – Intervention	Hypocalcaemia on admission (iCa <1.16 mmol/L)	N/A
C – Comparator	Normocalcaemia on admission (iCa ≥1.16 mmol/L)	N/A
O – Outcome	Primary outcome, the incidence of admission ionised hypocalcaemia.<br><br>Secondary outcomes include the association with physiological abnormalities:<br>▪ Haemodynamic instability<br>▪ Hyperkalaemia<br>▪ Hyperlactataemia<br>▪ pH abnormalities<br>▪ Coagulopathy<br><br>and adverse outcomes:<br>▪ Vasopressor requirement within 24 hours	N/A

For peer review

	▪ Transfusion requirement within 24 hours<br>▪ Activation of the major haemorrhage protocol within 24 hours<br>▪ Requiring invasive (operative or interventional radiology) intervention within 24 hours<br>▪ Hospital length of stay<br>▪ PICU length of stay<br>▪ Early mortality within 24 hours and medium mortality during episode of hospital admission (>24 hours) or within 30 days	
S – Study design	Clinical trials (randomised and non-randomised), observational studies (cohort and case-controlled) case reports, case series and literature reviews.	Systematic reviews.<br><br>Opinion articles.

For peer review only

References

1. Wolfe I, MacFarlane A, Donkin A, et al. Why children die: death in infants, children and young people in the UK. *Royal College of Paediatrics and Child Health.* 2014. Available online at: https://www.rcpch.ac.uk/sites/default/files/2017-06/Why%20children%20die%20-%20Death%20in%20infants%2C%20children%20and%20young%20people%20in%20the%20UK%20-%20Part%20A%202014-05.pdf (accessed 23rd October 2022)
2. Elbourne C, Cole E, Marsh S, et al. At risk child: a contemporary analysis of injured children in London and the South East of England: a prospective, multicentre cohort study. *BMJ Paediatr Open.* 2021;5(1):e001114. doi:10.1136/bmjpo-2021-001114
3. Davis JS, Satahoo SS, Butler FK, et al. An analysis of prehospital deaths: Who can we save? *J Trauma Acute Care Surg*. 2014;77(2):213-218. doi:10.1097/TA.0000000000000292
4. Moore EE, Moore HB, Kornblith LZ, et al. Trauma-induced coagulopathy [published correction appears in Nat Rev Dis Primers. 2022 Apr 22;8(1):25. *Nat Rev Dis Primers*. 2021;7(1):30. doi:10.1038/s41572-021-00264-3
5. Wray JP, Bridwell RE, Schauer SG, et al. The diamond of death: Hypocalcemia in trauma and resuscitation. *Am J Emerg Med*. 2021;41:104-109. doi:10.1016/j.ajem.2020.12.065
6. DeBot M, Sauaia A, Schaid T, Moore EE. Trauma-induced hypocalcemia. *Transfusion.* 2022;62 Suppl 1:S274-S280. doi:10.1111/trf.16959
7. Ditzel RM Jr, Anderson JL, Eisenhart WJ, et al. A review of transfusion- and trauma-induced hypocalcemia: Is it time to change the lethal triad to the lethal diamond? *J Trauma Acute Care Surg.* 2020;88(3):434-439. doi:10.1097/TA.0000000000002570
8. Brohi K, Gruen RL, Holcomb JB. Why are bleeding trauma patients still dying? *Intensive Care Med.* 2019;45(5):709-711. doi:10.1007/s00134-019-05560-x

9. Braasch MC, Turco LM, Cole EM, et al. The evolution of initial-hemostatic resuscitation and the void of posthemostatic resuscitation. *J Trauma Acute Care Surg*. 2020;89(3):597-601. doi:10.1097/TA.0000000000002576
10. Hamroun A, Pekar JD, Lionet A, et al. Ionized calcium: analytical challenges and clinical relevance. *J Lab Precis Med*. 2020; 5: 22. doi:10.21037/jlpm-20-60
11. Bilkovski RN, Cannon CM, Adhikari I, et al. Arterial and venous ionized calcium measurements: Is there a difference? *Ann Emerg Med*. 2004; 44 (4): S56. doi:10.1016/j.annemergmed.2004.07.183
12. Egi M, Kim I, Nichol A, et al. Ionized calcium concentration and outcome in critical illness. *Crit Care Med*. 2011;39:314–321. doi:10.1097/CCM.0b013e3181ffe23e
13. Mirzazadeh M, Morovat A, James T, et al. Point-of-care testing of electrolytes and calcium using blood gas analysers: it is time we trusted the results. *Emerg Med J*. 2016;33(3):181-186. doi:10.1136/emermed-2015-204669
14. Kronstedt S, Roberts N, Ditzel R, et al. Hypocalcemia as a predictor of mortality and transfusion. A scoping review of hypocalcemia in trauma and hemostatic resuscitation. *Transfusion*. 2022;62 Suppl 1(Suppl 1):S158-S166. doi:10.1111/trf.16965
15. Byerly, S., Inaba, K., Biswas, S, et al. Transfusion-Related Hypocalcemia After Trauma. *World journal of surgery*. 2020;44(11): 3743–3750. doi:10.1007/s00268-020-05712-x
16. Webster S, Todd S, Redhead J, et al. Ionised calcium levels in major trauma patients who received blood in the Emergency Department. *Emerg Med J*. 2016;33(8):569. doi: 10.1136/emermed-2015-205096
17. Cherry RA, Bradburn E, Carney DE, et al. Do early ionized calcium levels really matter in trauma patients? *J Trauma*. 2006;61(4):774-779. doi:10.1097/01.ta.0000239516.49799.63
18. Magnotti LJ, Bradburn EH, Webb DL, et al. Admission ionized calcium levels predict the need for multiple transfusions: a prospective study of 591 critically ill trauma patients. *J Trauma*. 2011;70(2):391-397. doi:10.1097/TA.0b013e31820b5d98
19. Vasudeva M, Mathew JK, Fitzgerald MC, et al. Hypocalcaemia and traumatic coagulopathy: an observational analysis. *Vox Sang*. 2020;115(2):189-195. doi:10.1111/vox.12875
20. Vasudeva M, Mathew JK, Groombridge C, et al. Hypocalcemia in trauma patients: A systematic review. *J Trauma Acute Care Surg*. 2021;90(2):396-402. doi:10.1097/TA.0000000000003027
21. Rossaint, R., Afshari, A., Bouillon, B. et al. The European guideline on management of major bleeding and coagulopathy following trauma: sixth edition. *Crit Care*. 2023; 27: 80. doi:10.1186/s13054-023-04327-7
22. Henry S. ATLS 10th edition offers new insights into managing trauma patients bulletin of the American College of Surgeons. *Am Coll Surg*. 2018: 56. Available online at: https://bulletin.facs.org/2018/06/atls-10th-edition-offers-new-insights-into-managing-traumapatients/#Chapter_3_Shock (accessed 23rd October 2022)
23. Barcelona SL, Thompson AA, Coté CJ. Intraoperative pediatric blood transfusion therapy: a review of common issues. Part I: hematologic and physiologic differences from adults; metabolic and infectious risks. *Paediatr Anaesth*. 2005;15(9):716–26. doi: 10.1111/j.1460-9592.2005.01548.x
24. Ciaraglia A, Lumbard D, Deschner B, et al. The Effects of Hypocalcemia in Severely Injured Pediatric Trauma Patients [published online ahead of print, 2023 Feb 13]. *J Trauma Acute Care Surg*. 2023;10.1097/TA.0000000000003902. doi:10.1097/TA.0000000000003902
25. Gimelraikh Y, Berant R, Stein M, et al. Early Hypocalcemia in Pediatric Major Trauma: A Retrospective Cohort Study [published online ahead of print, 2022 Apr 13]. *Pediatr Emerg Care*. 2022;10.1097/PEC.0000000000002719. doi:10.1097/PEC.0000000000002719
26. Epstein D, Ben Lulu H, Raz A, et al. Admission hypocalcemia in pediatric major trauma patients-An uncommon phenomenon associated with an increased need for urgent blood transfusion. *Transfusion*. 2022;62(7):1341-1346. doi:10.1111/trf.16936
27. Cornelius BG, Clark D, Williams B, et al. A retrospective analysis of calcium levels in pediatric trauma patients. *Int J Burns Trauma*. 2021;11(3):267-274. PMID: 34336394 PMCID: PMC8310872

28. Wolf J, Geneen LJ, Meli A, et al. Hyperkalaemia Following Blood Transfusion-a Systematic Review Assessing Evidence and Risks. *Transfus Med Rev*. 2022;36(3):133-142. doi:10.1016/j.tmrv.2022.04.003
29. Rishu AH, Khan R, Al-Dorzi HM, et al. Even mild hyperlactatemia is associated with increased mortality in critically ill patients. *Crit Care*. 2013;17(5):R197. doi:10.1186/cc12891
30. Samuels M, Wieteska E. Advanced Paediatric Life Support: A Practical Approach to Emergencies, 6th edn. Manchester, UK: *Advanced Paediatric Life Support Group*; New Jersey, USA: John Wiley & Sons Ltd; 2016. doi:10.1002/9781119241225
31. Linnaus ME, Notrica DM, Langlais CS, et al. Prospective validation of the shock index pediatric-adjusted (SIPA) in blunt liver and spleen trauma: An ATOMAC+ study. *J Pediatr Surg*. 2017;52(2):340-344. doi:10.1016/j.jpedsurg.2016.09.060
32. Nordin A, Coleman A, Shi J, et al. Validation of the age-adjusted shock index using pediatric trauma quality improvement program data [published online ahead of print, 2017 Oct 12]. *J Pediatr Surg.* 2017;S0022-3468(17)30645-0. doi:10.1016/j.jpedsurg.2017.10.023
33. Atkins D, Best D, Briss PA, et al. Grading quality of evidence and strength of recommendations. *BMJ*. 2004;328(7454):1490. doi:10.1136/bmj.328.7454.1490
34. Sterne JA, Hernán MA, Reeves BC, et al. ROBINS-I: a tool for assessing risk of bias in non-randomised studies of interventions. *BMJ.* 2016;355:i4919. doi:10.1136/bmj.i4919
35. Sterne JA, Sutton AJ, Ioannidis JP, et al. Recommendations for examining and interpreting funnel plot asymmetry in meta-analyses of randomised controlled trials. *BMJ*. 2011;343:d4002. doi:10.1136/bmj.d4002
36. Migliavaca CB, Stein C, Colpani V, et al. Meta-analysis of prevalence: I^2 statistic and how to deal with heterogeneity. *Res Synth Methods*. 2022;13(3):363-367. doi:10.1002/jrsm.1547

For peer review only

Online Supplementary Tables

Supplementary Table 1. CINAHL search strategy for a systematic review and meta-analysis exploring the incidence and associated outcomes with admission hypocalcaemia in paediatric major trauma

Search ID#	Search Terms	Last Run Via	Results from 3rd July 2023
**S29**	S25 AND S28	Interface - EBSCOhost Research Databases<br>Search Screen - Advanced Search<br>Database - CINAHL	2
**S28**	S26 OR S27	Interface - EBSCOhost Research Databases<br>Search Screen - Advanced Search<br>Database - CINAHL	95,090
**S27**	AB trauma	Interface - EBSCOhost Research Databases<br>Search Screen - Advanced Search<br>Database - CINAHL	77,482
**S26**	TI trauma	Interface - EBSCOhost Research Databases<br>Search Screen - Advanced Search<br>Database - CINAHL	39,417
**S25**	S23 OR S24	Interface - EBSCOhost Research Databases<br>Search Screen - Advanced Search<br>Database - CINAHL	11
**S24**	AB paediatric hypocalcaemia or pediatric hypocalcemia	Interface - EBSCOhost Research Databases<br>Search Screen - Advanced Search<br>Database - CINAHL	2
**S23**	TI paediatric hypocalcaemia or pediatric hypocalcemia	Interface - EBSCOhost Research Databases<br>Search Screen - Advanced Search<br>Database - CINAHL	9
**S22**	S3 AND S17	Interface - EBSCOhost Research Databases<br>Search Screen - Advanced Search<br>Database - CINAHL	351
**S21**	S3 AND S12	Interface - EBSCOhost Research Databases<br>Search Screen - Advanced Search<br>Database - CINAHL	0
**S20**	S18 OR S19	Interface - EBSCOhost Research Databases<br>Search Screen - Advanced Search<br>Database - CINAHL	19
**S19**	AB admission hypocalcaemia or admission hypocalcemia	Interface - EBSCOhost Research Databases<br>Search Screen - Advanced Search<br>Database - CINAHL	18

For peer review only

**S18**	TI admission hypocalcaemia or admission hypocalcemia	Interface - EBSCOhost Research Databases<br>Search Screen - Advanced Search<br>Database - CINAHL	1
**S17**	S15 OR S16	Interface - EBSCOhost Research Databases<br>Search Screen - Advanced Search<br>Database - CINAHL	835,597
**S16**	AB outcomes	Interface - EBSCOhost Research Databases<br>Search Screen - Advanced Search<br>Database - CINAHL	732,991
**S15**	TI outcomes	Interface - EBSCOhost Research Databases<br>Search Screen - Advanced Search<br>Database - CINAHL	213,098
**S14**	S3 AND S9 AND S12	Interface - EBSCOhost Research Databases<br>Search Screen - Advanced Search<br>Database - CINAHL	0
**S13**	S3 AND S6	Interface - EBSCOhost Research Databases<br>Search Screen - Advanced Search<br>Database - CINAHL	2
**S12**	S10 OR S11	Interface - EBSCOhost Research Databases<br>Search Screen - Advanced Search<br>Database - CINAHL	83
**S11**	AB normocalcaemia or normocalcemia	Interface - EBSCOhost Research Databases<br>Search Screen - Advanced Search<br>Database - CINAHL	78
**S10**	TI normocalcaemia or normocalcemia	Interface - EBSCOhost Research Databases<br>Search Screen - Advanced Search<br>Database - CINAHL	8
**S9**	S7 OR S8	Interface - EBSCOhost Research Databases<br>Search Screen - Advanced Search<br>Database - CINAHL	1,406
**S8**	AB hospital arrival	Interface - EBSCOhost Research Databases<br>Search Screen - Advanced Search<br>Database - CINAHL	1,349
**S7**	TI hospital arrival	Interface - EBSCOhost Research Databases<br>Search Screen - Advanced Search<br>Database - CINAHL	107
**S6**	S4 OR S5	Interface - EBSCOhost Research Databases<br>Search Screen - Advanced Search<br>Database - CINAHL	1,745
**S5**	AB hypocalcaemia	Interface - EBSCOhost Research Databases	1,451

	or hypocalcemia	Search Screen - Advanced Search Database - CINAHL	
**S4**	TI hypocalcaemia or hypocalcemia	Interface - EBSCOhost Research Databases Search Screen - Advanced Search Database - CINAHL	584
**S3**	S1 OR S2	Interface - EBSCOhost Research Databases Search Screen - Advanced Search Database - CINAHL	1,003
**S2**	AB paediatric trauma patients or pediatric trauma patients	Interface - EBSCOhost Research Databases Search Screen - Advanced Search Database - CINAHL	805
**S1**	TI paediatric trauma patients or pediatric trauma patients	Interface - EBSCOhost Research Databases Search Screen - Advanced Search Database - CINAHL	364

Supplementary Table 2. MEDLINE search strategy for a systematic review and meta-analysis exploring the incidence and associated outcomes with admission hypocalcaemia in paediatric major trauma

Search ID#	Search Terms	Last Run Via	Results from 3rd July 2023
S30	S26 AND S29	Interface - EBSCOhost Research Databases Search Screen - Advanced Search Database - MEDLINE	5
S29	S27 OR S28	Interface - EBSCOhost Research Databases Search Screen - Advanced Search Database - MEDLINE	276,599
S28	AB trauma	Interface - EBSCOhost Research Databases Search Screen - Advanced Search Database - MEDLINE	245,191
S27	TI trauma	Interface - EBSCOhost Research Databases Search Screen - Advanced Search Database - MEDLINE	87,100
S26	S24 OR S25	Interface - EBSCOhost Research Databases Search Screen - Advanced Search Database - MEDLINE	38
S25	AB paediatric hypocalcaemia or pediatric hypocalcemia	Interface - EBSCOhost Research Databases Search Screen - Advanced Search Database - MEDLINE	22
S24	TI paediatric hypocalcaemia or pediatric hypocalcemia	Interface - EBSCOhost Research Databases Search Screen - Advanced Search Database - MEDLINE	18

S23	S6 AND S22	Interface - EBSCOhost Research Databases<br>Search Screen - Advanced Search<br>Database - MEDLINE	4
S22	S3 AND S17	Interface - EBSCOhost Research Databases<br>Search Screen - Advanced Search<br>Database - MEDLINE	746
S21	S3 AND S12	Interface - EBSCOhost Research Databases<br>Search Screen - Advanced Search<br>Database - MEDLINE	0
S20	S18 OR S19	Interface - EBSCOhost Research Databases<br>Search Screen - Advanced Search<br>Database - MEDLINE	72
S19	AB admission hypocalcaemia or admission hypocalcemia	Interface - EBSCOhost Research Databases<br>Search Screen - Advanced Search<br>Database - MEDLINE	71
S18	TI admission hypocalcaemia or admission hypocalcemia	Interface - EBSCOhost Research Databases<br>Search Screen - Advanced Search<br>Database - MEDLINE	4
S17	S15 OR S16	Interface - EBSCOhost Research Databases<br>Search Screen - Advanced Search<br>Database - MEDLINE	2,179,197
S16	AB outcomes	Interface - EBSCOhost Research Databases<br>Search Screen - Advanced Search<br>Database - MEDLINE	2,053,450
S15	TI outcomes	Interface - EBSCOhost Research Databases<br>Search Screen - Advanced Search<br>Database - MEDLINE	450,999
S14	S3 AND S9 AND S12	Interface - EBSCOhost Research Databases<br>Search Screen - Advanced Search<br>Database - MEDLINE	0
S13	S3 AND S6	Interface - EBSCOhost Research Databases<br>Search Screen - Advanced Search<br>Database - MEDLINE	5
S12	S10 OR S11	Interface - EBSCOhost Research Databases<br>Search Screen - Advanced Search<br>Database - MEDLINE	870
S11	AB normocalcaemia or normocalcemia	Interface - EBSCOhost Research Databases<br>Search Screen - Advanced Search<br>Database - MEDLINE	837
S10	TI normocalcaemi	Interface - EBSCOhost Research Databases	52

	a or normocalcemia	Search Screen - Advanced Search<br>Database - MEDLINE	
S9	S7 OR S8	Interface - EBSCOhost Research Databases<br>Search Screen - Advanced Search<br>Database - MEDLINE	80,231
S8	AB hospital arrival or hospital admission	Interface - EBSCOhost Research Databases<br>Search Screen - Advanced Search<br>Database - MEDLINE	76,431
S7	TI hospital arrival or hospital admission	Interface - EBSCOhost Research Databases<br>Search Screen - Advanced Search<br>Database - MEDLINE	8,797
S6	S4 OR S5	Interface - EBSCOhost Research Databases<br>Search Screen - Advanced Search<br>Database - MEDLINE	11,987
S5	AB hypocalcaemia or hypocalcemia	Interface - EBSCOhost Research Databases<br>Search Screen - Advanced Search<br>Database - MEDLINE	10,707
S4	TI hypocalcaemia or hypocalcemia	Interface - EBSCOhost Research Databases<br>Search Screen - Advanced Search<br>Database - MEDLINE	3,153
S3	S1 OR S2	Interface - EBSCOhost Research Databases<br>Search Screen - Advanced Search<br>Database - MEDLINE	2,051
S2	AB paediatric trauma patients or pediatric trauma patients	Interface - EBSCOhost Research Databases<br>Search Screen - Advanced Search<br>Database - MEDLINE	1,764
S1	TI paediatric trauma patients or pediatric trauma patients	Interface - EBSCOhost Research Databases<br>Search Screen - Advanced Search<br>Database - MEDLINE	649

Supplementary Table 3. EMBASE search strategy for a systematic review and meta-analysis exploring the incidence and associated outcomes with admission hypocalcaemia in paediatric major trauma

#	Query	Results from 3rd July 2023
1	(paediatric trauma patients or pediatric trauma patients).m_titl.	384
2	(paediatric trauma patients or pediatric trauma patients).mp. [mp=title, abstract, heading word, drug trade name, original title, device manufacturer, drug manufacturer, device trade name, keyword heading word, floating subheading word, candidate term word]	1,172

3	limit 2 to abstracts	1,155
4	1 or 3	1,172
5	(hypocalcaemia or hypocalcemia).m_titl.	3,811
6	(hypocalcaemia or hypocalcemia).mp. [mp=title, abstract, heading word, drug trade name, original title, device manufacturer, drug manufacturer, device trade name, keyword heading word, floating subheading word, candidate term word]	31,406
7	limit 6 to abstracts	26,928
8	5 or 7	27,890
9	(hospital arrival or hospital admission).m_titl.	4,381
10	(hospital arrival or hospital admission).mp. [mp=title, abstract, heading word, drug trade name, original title, device manufacturer, drug manufacturer, device trade name, keyword heading word, floating subheading word, candidate term word]	292,976
11	limit 10 to abstracts	245,800
12	9 or 11	246,388
13	(normocalcaemia or normocalcemia).m_titl.	63
14	(normocalcaemia or normocalcemia).mp. [mp=title, abstract, heading word, drug trade name, original title, device manufacturer, drug manufacturer, device trade name, keyword heading word, floating subheading word, candidate term word]	1,223
15	limit 14 to abstracts	1,213
16	13 or 15	1,223
17	4 and 8 and 12	1
18	4 and 8	6
19	(paediatric hypocalcaemia or pediatric hypocalcemia).m_titl.	2
20	(paediatric hypocalcaemia or pediatric hypocalcemia).mp. [mp=title, abstract, heading word, drug trade name, original title, device manufacturer, drug manufacturer, device trade name, keyword heading word, floating subheading word, candidate term word]	4
21	limit 20 to abstracts	2
22	19 or 21	4
23	(admission hypocalcaemia or admission hypocalcemia).m_titl.	2
24	(admission hypocalcaemia or admission hypocalcemia).mp. [mp=title, abstract, heading word, drug trade name, original title, device manufacturer, drug manufacturer, device trade name, keyword heading word, floating subheading word, candidate term word]	11
25	limit 24 to abstracts	11
26	23 or 25	11
27	4 and 16	0

For peer review only

PRISMA-P (Preferred Reporting Items for Systematic review and Meta-Analysis Protocols) 2015 checklist: recommended items to address in a systematic review protocol*

Section and topic	Item No	Checklist item	
**ADMINISTRATIVE INFORMATION**			
Title:			
Identification	1a	Identify the report as a protocol of a systematic review	Page 1
Update	1b	If the protocol is for an update of a previous systematic review, identify as such	
Registration	2	If registered, provide the name of the registry (such as PROSPERO) and registration number	Page 1
Authors:			
Contact	3a	Provide name, institutional affiliation, e-mail address of all protocol authors; provide physical mailing address of corresponding author	Page 1
Contributions	3b	Describe contributions of protocol authors and identify the guarantor of the review	
Amendments	4	If the protocol represents an amendment of a previously completed or published protocol, identify as such and list changes; otherwise, state plan for documenting important protocol amendments	N/A
Support:			
Sources	5a	Indicate sources of financial or other support for the review	
Sponsor	5b	Provide name for the review funder and/or sponsor	Page 1
Role of sponsor or funder	5c	Describe roles of funder(s), sponsor(s), and/or institution(s), if any, in developing the protocol	
**INTRODUCTION**			
Rationale	6	Describe the rationale for the review in the context of what is already known	Page 2
Objectives	7	Provide an explicit statement of the question(s) the review will address with reference to participants, interventions, comparators, and outcomes (PICO)	Page 3
**METHODS**			
Eligibility criteria	8	Specify the study characteristics (such as PICO, study design, setting, time frame) and report characteristics (such as years considered, language, publication status) to be used as criteria for eligibility for the review	Page 3
Information sources	9	Describe all intended information sources (such as electronic databases, contact with study authors, trial registers or other grey literature sources) with planned dates of coverage	Page 3
Search strategy	10	Present draft of search strategy to be used for at least one electronic database, including planned limits, such that it could be repeated	Page 3
Study records:			
Data management	11a	Describe the mechanism(s) that will be used to manage records and data throughout the review	Page 3

Selection process	11b	State the process that will be used for selecting studies (such as two independent reviewers) through each phase of the review (that is, screening, eligibility and inclusion in meta-analysis)	
Data collection process	11c	Describe planned method of extracting data from reports (such as piloting forms, done independently, in duplicate), any processes for obtaining and confirming data from investigators	Page 3
Data items	12	List and define all variables for which data will be sought (such as PICO items, funding sources), any pre-planned data assumptions and simplifications	
Outcomes and prioritization	13	List and define all outcomes for which data will be sought, including prioritization of main and additional outcomes, with rationale	Page 3
Risk of bias in individual studies	14	Describe anticipated methods for assessing risk of bias of individual studies, including whether this will be done at the outcome or study level, or both; state how this information will be used in data synthesis	Page 3
Data synthesis	15a	Describe criteria under which study data will be quantitatively synthesised	
	15b	If data are appropriate for quantitative synthesis, describe planned summary measures, methods of handling data and methods of combining data from studies, including any planned exploration of consistency (such as I^2, Kendall's τ)	Page 4
	15c	Describe any proposed additional analyses (such as sensitivity or subgroup analyses, meta-regression)	
	15d	If quantitative synthesis is not appropriate, describe the type of summary planned	
Meta-bias(es)	16	Specify any planned assessment of meta-bias(es) (such as publication bias across studies, selective reporting within studies)	Page 4
Confidence in cumulative evidence	17	Describe how the strength of the body of evidence will be assessed (such as GRADE)	Page 4

*** It is strongly recommended that this checklist be read in conjunction with the PRISMA-P Explanation and Elaboration (cite when available) for important clarification on the items. Amendments to a review protocol should be tracked and dated.** **The copyright for PRISMA-P (including checklist) is held by the PRISMA-P Group and is distributed under a Creative Commons Attribution Licence 4.0.**

From: Shamseer L, Moher D, Clarke M, Ghersi D, Liberati A, Petticrew M, Shekelle P, Stewart L, PRISMA-P Group. Preferred reporting items for systematic review and meta-analysis protocols (PRISMA-P) 2015: elaboration and explanation. BMJ. 2015 Jan 2;349(jan02 1):g7647.

For peer review only
